# Rhodococcus parequi sp. nov., a new species isolated from equine farm soil closely related to the pathogen Rhodococcus equi

**DOI:** 10.1099/ijsem.0.006679

**Published:** 2025-03-10

**Authors:** José A. Vazquez-Boland, Jorge Val-Calvo, Fabien Duquesne, Francesca Decorosi, Carlo Viti, Sandrine Petry, Mariela Scortti

**Affiliations:** 1 Microbial Pathogenomics Laboratory, Edinburgh Medical School (Biomedical Sciences), University of Edinburgh, Edinburgh, UK; 2 ANSES, Laboratory for Animal Health, Physiopathology and Epidemiology of Equine Diseases Unit, Goustranville, France; 3 Department of Agriculture, Food, Environment and Forestry, University of Florence, Florence, Italy

**Keywords:** genus *Rhodococcus*, *Rhodococcus equi*, *Rhodococcus parequi*, *Rhodococcus* phylogenomics, *Rhodococcus* systematics, *Rhodococcus* taxonomy

## Abstract

We present the description of the new species, *Rhodococcus parequi*, found during phylogenomic investigations of a global collection of strains identified as *Rhodococcus* (*Prescottella*) *equi*. Strain PAM 2766^T^ was isolated from horse-breeding farm soil in Normandy, France, and was indistinguishable from *R. equi* based on the usual identification tests. Whole-genome phylogenetic analyses located PAM 2766^T^ in the same *Rhodococcus* sublineage as *R. equi*, together with *Rhodococcus agglutinans*, *Rhodococcus defluvii*, *Rhodococcus soli*, *Rhodococcus subtropicus*, *Rhodococcus spongiicola* and *Rhodococcus xishaensis*. PAM 2766^T^ is most closely related to, but sufficiently distinct from, *R. equi* DSM 20307 ^T^ to be considered a separate species. The average nt identity (ANI) and average aa identity (AAI) values are 88.60% and 92.35, respectively, well below the species cutoff. The PAM 2766^T^ draft genome is ~5.3 Mb in size with 68.98% G+C mol content. PAM 2766^T^ is strictly aerobic and non-motile and produces smooth, creamy to buff-coloured colonies very similar to those of *R. equi*. It phenotypically differs from the latter by the ability to grow at 5 °C, a strongly positive urease test at 24 h and specificities in the carbon and nitrogen source utilization profile as determined by phenotype microarray screens. Our data indicate that PAM 2766^T^ belongs to a novel species, for which the name *Rhodococcus parequi* sp. nov. is proposed. *R. parequi* was avirulent in macrophage infection assays and is assumed to be non-pathogenic. The type strain is PAM 2766^T^ (=CETC 30995^T^=NCTC 14987^T^).

## Introduction

The bacterial genus *Rhodococcus* Zopf 1891 (Approved Lists 1980) [1,2], within the *Nocardiaceae* family of the *Mycobacteriales*, comprises a large group of irregular Gram-positive, aerobic coccobacilli with 51 recognized species and an ever-growing number of unclassified isolates (https://www.bacterio.net) [3]. The rhodococci are ubiquitous saprophytes that can be isolated from a wide variety of habitats including soil, waters, marine sediments and extreme environments such as deep sea, caves and xenobiotic-contaminated sites [4,6]. One member of the genus, *Rhodococcus equi*, commonly found in soil, is a major equine pathogen and human opportunistic pathogen [7,8]. This study reports the isolation of a novel *Rhodococcus* species that is closely related to *R. equi*, with potential implications in the identification of this pathogen and in studies on its ecology, epidemiology and environmental distribution. Here, we use the name *R. equi* instead of the synonym *Prescottella equi* (Magnusson 1923) Sangal *et al*. 2022 [9], and the circumscription of the genus *Rhodococcus* Zopf 1891 as emended by Val-Calvo and Vázquez-Boland 2023 [10]. This emendation was proposed in a recent study that examined the *Mycobacteriales* taxonomy using a novel phylogenomic approach for objective genus demarcation based on distance-normalized tree clustering and network analysis of genomic relatedness indices [11]. The study concluded that the creation of a nested genus *Prescottella* within the *Rhodococcus* monophyletic radiation was unwarranted and an example of genus oversplitting (a current trend stemming from the arbitrary application of genomic-based demarcation metrics) [11].

## Strain isolation

Strain PAM 2766^T^ derives from a culture kept in the Vazquez-Boland’s laboratory collection obtained in 1999 from a soil sample from a stud farm in Normandy, France. The original bacterial culture was initially classified as *R. equi* based on colony morphology, API Coryne (BioMériux) biochemical profiling, detection of cholesterol oxidase activity by a CAMP (Christie–Atkins–Munch-Petersen test of synergistic haemolysis on sheep blood agar with sphingomyelinase C-producing indicator bacteria)-like assay with *Listeria ivanovii* [12,13] and a positive PCR for the *R. equi* cholesterol oxidase gene *choE* (considered a species-specific identification marker [14,17]). In a previous study, the culture (identified as PAM 1352) was found to lack the *R. equi* virulence plasmid by TRAVAP typing, a PCR-based method to differentiate the three *R. equi* virulence plasmid types [16,18]. During a population phylogenomic study of the *R. equi* species, whole-genome sequencing (WGS) analysis suggested that ‘*R. equi*’ PAM 1352 was contaminated with a closely related taxonomically unidentified micro-organism. Meticulous re-isolation followed by WGS confirmed that PAM 1352 was a mixed culture of two different bacteria with very similar colonial morphologies: one corresponded to *R. equi* and the other to a putative new species. The pure culture of the latter was assigned PAM number 2766 and deposited at the NCTC (National Collection of Type Cultures) repository under number 14987 and CECT [Colección Española de Cultivos Tipo (Spanish Type Culture Collection)] repository under number 30995 (henceforth PAM 2766^T^, NCTC 14987^T^ and CECT 30995^T^, respectively).

## Genomic characterization

Genomic DNA of PAM 2766^T^ was isolated (GenElute™ Bacterial Genomic DNA, Sigma) and sequenced by Illumina in a NovaSeq X Plus PE150 platform (Novogene UK Ltd.). A total of 9 million 150 bp paired-end reads were obtained, representing an average coverage depth of 278×. The raw sequencing data were processed using FastQC (https://www.bioinformatics.babraham.ac.uk/projects/fastqc/) and Trimmomatic [19] (settings: Leading 3, Trailing 3, Slidingwindow 4:15 and Minlen 36) to remove low-quality reads and adapter sequences and then assembled using SPAdes v3.15.2 [20] (option isolate active, *k*-mer length and read coverage set to auto). The draft PAM 2766^T^ genome consisted of 16 contigs of >500 bp with a total length of 5 295 699 bp and a G+C content of 68.98 mol%. Contig scores of N50=611 300 and L50=4 determined using QUAST v5.1.0rc1 [21] were consistent with a quality assembly for taxonomic purposes. The PAM 2766^T^ draft genome was annotated using the NCBI Prokaryotic Genome Annotation Pipeline (PGAP) (2023-10-03.build7061) [22].

When aligning in QUAST the unfractionated PAM 2766^T^ genome contigs to the 103S reference genome (the first complete and only manually curated genome for *R. equi*; GenBank accession no. FN563149.1) [23], several metrics suggested that it deviated from a typical *R. equi* genome assembly. For example, the genome fraction % (percentage of aligned contig bases in the reference genome) and total aligned length (number of aligned contig bases in the assembly) were 1.04% and 53 807 for PAM 2766^T^ vs 95.23% and 4 804 699 for the *R. equi* DSM 20307^T^ genome, respectively. Similar diverging values were obtained with other random *R. equi* genome assemblies.

**Fig. 1.**
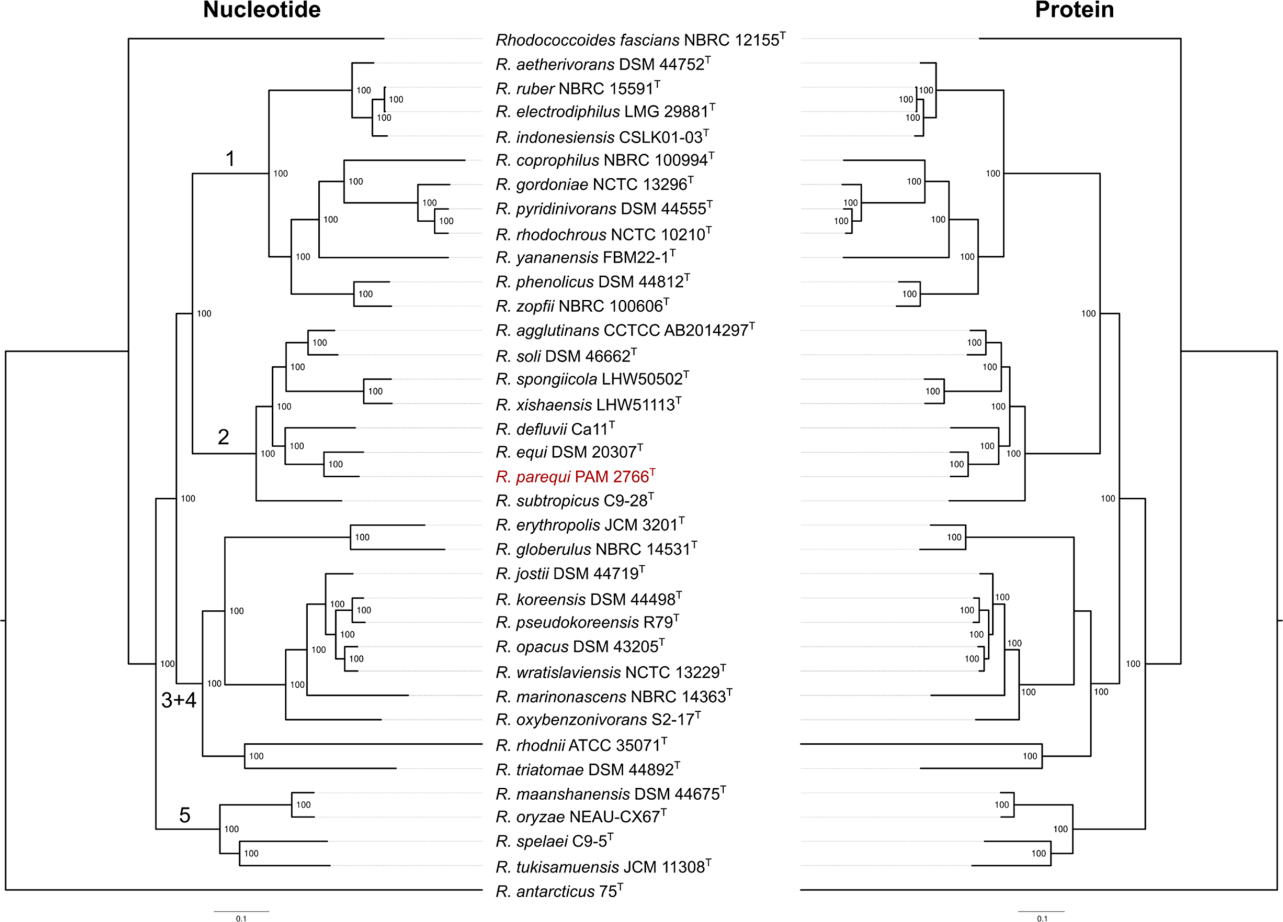
Core-genome maximum-likelihood (ML) trees based on concatenated sequence alignments of conserved genes (left, 409 gene markers) or proteins (right, 323 protein markers). The analysis includes the type strains of all *Rhodococcus* species with genome assemblies available at the NCBI as of January 2024 (acc. nos. in Table S1, available in the online Supplementary Material). *Rhodococcoides fascians* from an early diverging branch of the rhodococcal radiation [11] was used as an outgroup. The tree was midpoint rooted because of the uncertain position of *Rhodococcus antarcticus* 75^T^ [53] within the genus *Rhodococcus*. Branch support is given for 1000 ultrafast bootstrap replicates (IQtree UFBoot). The large case numbers on the nt-based tree indicate the main *Rhodococcus* sublineages as defined in ref. [11]. The trees are entirely congruent with our previous ML phylogenies based on *Mycobacteriales* or *Nocardiaceae* core-genome alignments (the latter incorporating a larger strain representation than the type’s) [11]. The only exception is that *Rhodococcus rhodnii* ATCC 33071^T^ and *Rhodococcus triatomae* DSM 44892^T^ form here an independent branch at the root of the monophyletic line of descent containing sublineages 3 and 4 instead of being part of the sublineage 4 radiation (see ref. [11]).

The genomic distinctiveness of PAM 2766^T^ was confirmed using two overall genomic relatedness indices recognized as robust indicators for species demarcation, i.e. the average nt identity (ANI) and average aa identity (AAI) [22,24,26]. The values for both indices between PAM 2766^T^ and *R. equi* DSM 20307^T^ were well below the <95% species definition cutoff [25] (ANI=88.60±4.11; AAI=92.35±7.42). Digital DNA–DNA hybridization (dDDH) analyses performed using the Genome-to-Genome Distance Calculator (GGDC) 3.0 [27] also reported a low probability (1.4%) for a dDDH value greater than the species delimitation threshold of >70% [24,27] (Table 1). Next in relatedness were the *Rhodococcus* species located within the same phylogenomic sublineage as *R. equi* (rhodococcal sublineage no. 2 [11]) (Fig. 1). ANI/AAI values ranged from 85.21/85.80 for *Rhodococcus agglutinans* to 82.00/81.32, 82.13/80.73 and 82.83/80.87 for *Rhodococcus xishaensis*, *Rhodococcus spongiicola* and *Rhodococcus subtropicus*, respectively (Table 1). Note that *Rhodococcus soli* DSM 46662^T^ was *de novo* sequenced (GenBank accession no. JBDLNU000000000) as its genome information was not publicly available at the time of this study.

**Table 1.** Genome relatedness index comparisons between *R. parequi* PAM 2766^T^ and most closely related species (*Rhodococcus* phylogenomic sublineage no. 2 [11])

	16S*(% similarity)	ANI†	AAI†	dDDH-GGD‡(% probability)
*R. agglutinans* CFHS 0262^T^	99.03	85.21±5.19	85.80±11.48	28.20%–0.1522 (0.05)
*Rhodococcus defluvii* Ca11^T^	98.89	84.52±5.13	85.31±11.68	27.60%–0.1565 (0.03)
*R. equi* DSM 20307^T^	100§	88.60±4.11	92.35±7.42	37.60%–0.1079 (1.44)
*R. soli* DSM 46662^T^	99.31	84.57±5.08	84.95±12.23	27.70%–0.1558 (0.04)
*R. spongiicola* LHW50502^T^	97.15	82.13±4.65	80.73±14.12	24.10%–0.1808 (0.01)
*R. subtropicus* C9-28^T^	98.68	82.83±4.94	80.87±13.30	24.70%–0.1763 (0.01)
*R. xishaensis* LHW51113^T^	97.15	82.00±4.63	81.32±13.48	24.20%–0.1804 (0.01)

* Determined using EzBioCloud 16S database [54], except for *R. equi* (see footnote §).

† Calculated using the scripts ani.rb and aai.rb from the ‘Enveomics collection’ [55].

‡ dDDH and genome-to-genome distance (GGD) calculated using GGDC 3.0 based on the recommended formula 2 [identities/high-scoring segment pair (HSP) length] for incomplete/draft genomes [27]. In parentheses, the probability of a dDDH >70% (i.e. same species) by logistic regression.

§ Based on the 16 rDNA sequence of DSM 20307T genome assembly GenBank accession no. LWTX01000024 (LWTX01000000 – contig024) [29]. The sequence from EzBioCloud for this same strain (accession no. AF490539.1) contains two errors (see text).

These data indicated that PAM 2766^T^ was genomically sufficiently distinct from all the species within the rhodococcal monophyletic sublineage containing *R. equi* to warrant separate species status.

## 16S rRNA gene sequence

The 16S rRNA gene sequence of PAM 2766^T^ [from both the draft genome assembly and a 16S rRNA gene PCR amplicon obtained using oligonucleotide primers 27F 5′-AGAGAGTTTGATCCTGGCTGGCTCAG-3′ and 1492R 5′-CGGCTACCTACCTTGTTACGACTT-3′ [28] and high-fidelity DNA polymerase Q5 (New England Biolabs)] was found to be 100% identical to that of *R. equi*. This was determined using a high-quality draft genome assembly of *R. equi* DSM 20307^T^ from our laboratory [GenBank accession no. LWTX01000024 (LWTX01000000 – contig024) [29]] (Table S1) as well as two other *R. equi* 16S rRNA gene sequences available in the databases, GenBank accession nos. X80614 for DSM 20307^T^ deposited in 1995 [30] and FJ468344 for ATCC 6939^T^ deposited in 2008. Additionally, only three of the six other species encompassed in the same rhodococcal sublineage as *R. equi* (the most distantly related on the basis of ANI/AAI, i.e. *R. subtropicus*, *R. spongiicola* and *R. xishaensis*) had 16S rRNA gene similarity scores below the 98.7% species demarcation standard [22,25, 26, 31] (Table 1). It is not unusual among closely related rhodococci to share virtually identical 16S rRNA gene sequences [32,35], highlighting the limitations of the 16S rRNA gene sequences for accurate differentiation of prokaryotic species [25,36, 37].

Of note, the 16S rRNA gene sequence similarity dropped to 99.86% when using GenBank accession no. AF490539, designated as the reference sequence for *R. equi* DSM 20307^T^ by major databases [EzBioCloud 16S database (https://www.ezbiocloud.net/db) [38] and LPSN (https://lpsn.dsmz.de/species/rhodococcus-equi) [3]]. Since the 16S rRNA gene sequences from >100 *R. equi* WGSs (including a reference diversity set of 27 previously characterized isolates from different sources [29]; NCBI BioProject PRJNA316970]) showed all 100% similarity to LWTX01000024 (as well as X80614 and FJ468344), a caveat must be sounded as to the inaccuracy of the AF490539 sequence. AF490539 appears to contain two sequencing errors: a missing cytosine (of a series of four cytosines) between C50 and T51 and a missing guanine (of a series of five guanines) between C53 and G54.

## Phylogenomic analysis

To determine the phylogenetic position and relationships of PAM 2766^T^, we generated a core-genome phylogeny using all available genome assemblies for the type strains of each species of the genus *Rhodococcus* (as emended by Val-Calvo and Vázquez-Boland 2023 [11,39]) with status name ‘correct’ in the LPSN repository (https://lpsn.dsmz.de/genus/rhodococcus, accessed January 2024) (Table S1). The *Rhodococcoides* gen. nov. [11] circumscription, which forms a distinct, early diverging branch at the base of the rhodococcal radiation [11,29], was excluded from the phylogeny except for the type species, *Rhodococcoides fascians* (Tilford 1936) Val-Calvo and Vázquez-Boland 2024 [basonym: ‘*Phytomonas fascians*’ Tilford 1936; homotypic synonym: *Rhodococcus fascians* (Tilford 1936) Goodfellow 1984] NBRC 12155^T^, which was used as an outgroup. Using a closely related outgroup maximizes the number of core genomic markers and thus the resolution of the phylogenetic reconstruction. Core-genome orthologous genes were identified using the Get-Homologues package v22082022 [40] (Clusters of Orthologous Groups[COG] and OrthoMCL Markov clustering algorithms output intersect with the following settings: minimal coverage 75% maximum *E*-value 1e−05) and filtered to exclude recombinant alignments or alignments producing anomalous or poorly supported trees using the Get-Phylomarkers v2.4.6 tool [41]. Concatenated alignments based on the nt sequence of conserved coding sequence (409 gene markers) and the aa sequence of conserved gene products (323 protein markers) were generated (Fig. 1). Maximum-likelihood (ML) phylogenetic trees were built using IQtree2 software v2.2.0 [42] with the substitution models GTR+F+ASC+R5 and LG+F+R3A for the nt and protein sequences, respectively.

**Fig. 2.**
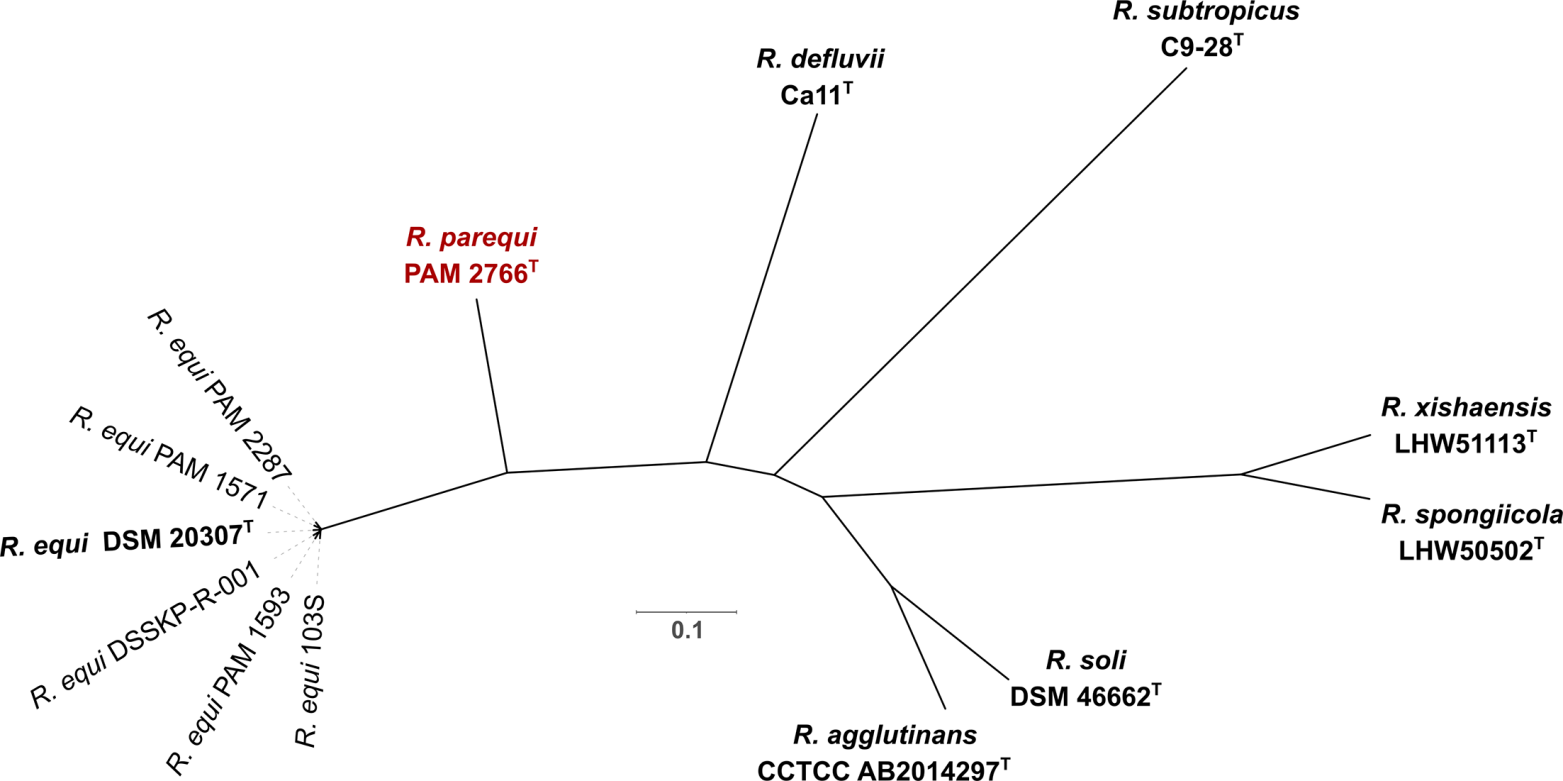
Unrooted core-genome ML tree of *Rhodococcus* monophyletic sublineage no. 2 containing *R. equi* and *R. parequi* PAM 2766^T^. The analysis includes a selection of *R. equi* strains representative of the genomic diversity of the species [29] (acc. nos. in Table S1). Based on a concatenated alignment of 828 core genes identified using Get-Homologues v22082022 [40] (orthologues identified with the COG and OrthoMCL clustering algorithms intersect using the settings minimal coverage 75% and maximum *E*-value 1e−05). Substitution model was GTR+F+ASC+R3. Branch support for each node is 100 (1000 ultrafast bootstrap replicates using IQtree UFBoot).

The resulting nt- and protein sequence-based *Rhodococcus* genus ML trees were strongly supported and fully consistent with each other (Fig. 1). Both phylogenies located PAM 2766^T^ in rhodococcal sublineage no. 2 [11] as the most closely related species to *R. equi*. The evolutionary distance between PAM 2766^T^ and *R. equi* is equivalent to or greater than that between other pairs of closely related species within other *Rhodococcus* sublineages (e.g. *Rhodococcus rhodochrous* and *Rhodococcus pyridinivorans* in sublineage 1, or *Rhodococcus opacus* and *Rhodococcus wratislaviensis* in sublineage 4 [11]) (Fig. 1), also supporting the classification of PAM 2766^T^ as a distinct species. This is most clearly illustrated by a core-genome ML tree of sublineage 2 *Rhodococcus* spp. in which several *R. equi* isolates representative of the diversity of the species were included for reference (Fig. 2). Here, whilst the *R. equi* isolates form a punctiform radiation due to their extremely short relative genetic distances [29], PAM 2766^T^ branches out at a significant distance in a comparable fashion to the other species encompassed in this rhodococcal sublineage. The tree also shows that PAM 2766^T^ represents the most closely related taxon to *R. equi* in the *Rhodococcus* circumscription (Fig. 2).

## Phenotypic characterization

PAM 2766^T^ are Gram-positive, non-spore-forming coccobacilli morphologically similar to *R. equi* and display the general biochemical/physiological profile of the genus *Rhodococcus*. This includes strictly aerobic growth [tested in an anaerobic chamber with AnaeroGen™ sachet (Thermo Fisher) at 30 °C for 20 days], non-motility [tested in tryptic soy broth (TSB) 0.3% agar incubated at 30 °C for 14 days] and positive catalase and oxidase negative reactions. The growth characteristics are akin to those of *R. equi*, both species forming on tryptic soy agar (TSA) smooth, creamy, buff-coloured, shiny colonies that are difficult to differentiate from each other. Grading of growth intensity/abundance in different media was TSB (VWR Chemicals 84675)>brain heart infusion (BHI) (VWR Chemicals 84626)>Luria–Bertani (LB) (Sigma L3022) based on growth curves in an Optima BMG plate reader (Fig. S2).

Also similar to *R. equi* [23], PAM 2766^T^ requires thiamine supplementation for growth (Fig. S3A). Genomic analysis of *R. equi* identified the disruption of the *thiCD* locus in the thiamine biosynthesis pathway, caused by a horizontal gene acquisition event with concomitant deletion of the *thiC* gene, as the likely cause of the thiamine auxotrophy [23,29] (Fig. S3B). Inspection of the genetic structure of the homologous region in PAM 2766^T^ showed that it was virtually identical to that of *R. equi*, including the same *thiCD* genomic lesion (Fig. S3B). Since species with a complete *thiCD* locus are also present in the *Rhodococcus* radiation [e.g. *Rhodococcus erythropolis* [23] (Fig. S3B)], the *thiCD* disruption/*thiC* deletion likely took place somewhere between the common ancestor of the genus and that of the rhodococcal sublineage encompassing *R. equi* and PAM 2766^T^. The thiamine auxotrophy of *R. equi* has been seen as an adaptive trait to the natural reservoirs of this species, manure-rich soil and the large intestine, where microbiota-derived thiamine is likely to be readily accessible [8]. We surmise that a similar explanation may apply to PAM 2766^T^.

A search for phenotypic differentiation markers found that PAM 2766^T^ can grow at 5 °C, evident after 10 days of incubation*,* in contrast to *R. equi* DSM 20307^T^ [and the *R. equi* reference genome strain 103S (PAM 1126) as a representative of the other major phylogenomic subdivision of the species [29]] (Table 2). PAM 2766^T^ also gave a clearly positive urease test after 24 h at 30 °C, whilst *R. equi* required a longer incubation (minimum of 48 h) and the reaction was generally weaker (Table 2). It is worth noting that the urease test was in all cases negative using the API Coryne strips (BioMériux). The reason for the discrepancy is that in the API Coryne gallery, urease activity is tested under anaerobic conditions, preventing the growth of the strictly aerobic *Rhodococcus* bacteria.

**Table 2.** Main phenotypic characteristics of *R. parequi* PAM 2766^T^ and *R. equi.*

	*R. parequi*PAM 2766^T^	*R. equi*DSM 20307^T^	*R. equi* 103S(PAM 1126)
Growth temperature range (°C)*	5†−45	10–45‡	10–45
pH range‡	5–10	5–10	5–10
NaCl tolerance range (w/v)§	1–3	1–4¶	1–4
Urease** - 24 h	+	w	−
- 48 h	+	+	w
API Coryne profile	1110004	1110004	1110004
Phenotype MicroArray™			
C-sources			
Tween 20	−	+	+
4-Hydroxybenzoic acid	−	+	+
*β*-Hydroxybutyric acid	−	+	+
N-sources			
l-Cysteine	−	+	+
l-Lysine	−	+	+
l-Citrulline	−	+	+
l-Ornithine	−	+	+
Thymine	−	+	+
*ε*-Amino-*N*-caproic acid	−	+	+
*δ*-Amino-*N*-valeric acid	−	+	+

* Tested on TSA plates.

† Growth at 5 °C was observed after 10 days.

‡ Differs from emendation of *R. equi* (Magnusson 1923) Goodfellow and Alderson 1977 by Goodfellow *et al*. [56] where a temperature growth range of 5–40 °C was reported.

§ Determined using growth curve assays in TSB at 30 °C using an Optima BMG plate reader (48-well plates with 400 µl medium/well, 200 r.p.m. shaking, readings every 30 min). The pH of the medium was adjusted at 1.0 intervals with phosphate (pH 2.0–7.0), Tris-HCl (pH 7.0–9.0) or sodium bicarbonate (pH 9.0–11.0). NaCl concentrations between 0 and 10% (w/v) were tested at 1% intervals.

¶ Differs from Lee *et al*. [6], in which *R. equi* DSM 20307T is reported to grow on 5% NaCl.

** Determined using Christensen’s urea agar as per ASM protocols (https://asm.org/getattachment/ac4fe214-106d-407c-b6c6-e3bb49ac6ffb/urease-test-protocol-3223.pdf) modified by replacing dextrose (which *R. equi* does not use, or uses inefficiently) with 25 mM sodium lactate (preferred carbon source that promotes vigorous growth of *R. equi* [29]).

Results: +, positive; w, weakly positive; −, negative. See table footnotes for methods and Fig. 4 for an overview of Biolog’s Phenotype MicroArray™ results. All three strains exhibited thiamine-dependent growth (Fig. S3A) and a positive CAMP-like synergistic haemolysis reaction (tested in Columbia 5% sheep blood agar using an indicator strain of *L. ivanovii* as previously described [13]).

**Fig. 3.**
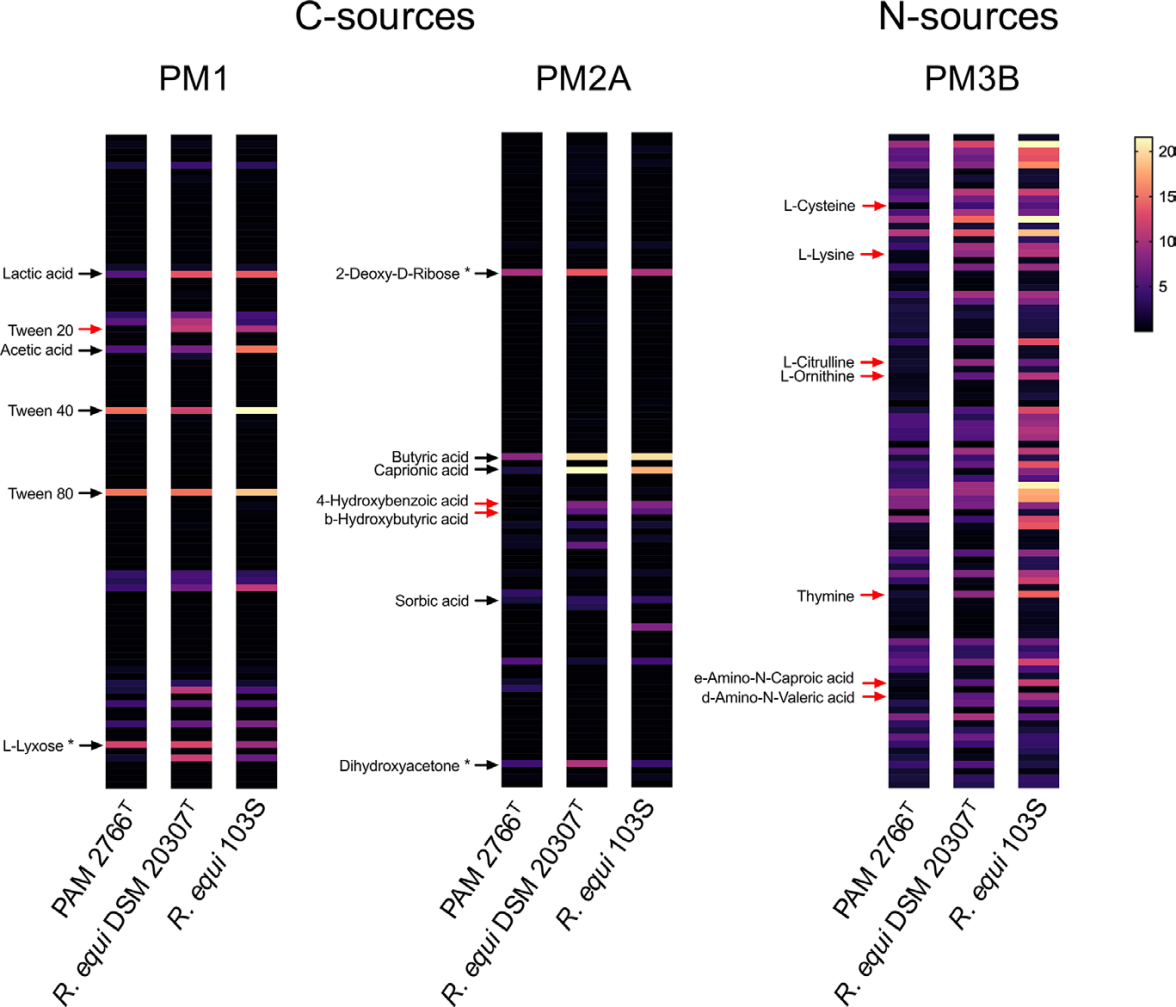
Heat map of Phenotype MicroArray™ (PM) results for carbon and nitrogen source utilization by PAM 2766^T^, *R. equi* DSM 20307^T^ and *R. equi* 103S (PAM 1126). Bacterial inocula were grown at 30 °C in TSB until the stationary phase and then suspended in *R. equi* mineral medium [57] (modified as in refs. [23,29]; mReMM) and transferred to the PM plates. Incubation was performed at 30 °C with OD_590_ monitored every 15 min for 48 h in an OmniLog reader. Strains were tested in duplicate and results were analysed using OmniLog software. Maximum growth is represented in graded colours from lowest (black) to highest (yellow). Red arrows indicate differential utilization of a substrate between PAM 2766^T^ and *R. equi.* Black arrows in the PM1 and PM2A plates indicate a carbon source utilized by the three tested bacteria (see Table S2 for detailed results). Asterisks indicate false positive reactions in the PM2A plate previously reported in ref. [23].

Phenotype MicroArray™ (Biolog Inc.) screens for carbon (PM1 and PM2A plates) and nitrogen (PM3B plates) sources were used to identify additional differential phenotypic markers. PAM 2766^T^ utilized, with different efficiency, 26 out of the 190 tested carbon sources. Like *R. equi* [23], PAM 2766^T^ seems to assimilate carbon mainly through lipid metabolism, growing vigorously on different organic acids as a sole carbon source, including lactate, acetate and sorbate (Fig. 3 and Table S2). PAM 2766^T^ could be differentiated from *R. equi* by the inability to grow on Tween 20 (polyoxyethylene sorbitan monolaurate), 4-hydroxybenzoic acid and *β*-hydroxybutyric acid. Tween 20 has been reported to be toxic for some *Rhodococcus* spp. whilst it is utilized by other rhodococci such as *Rhodococcus jostii* or *R. opacus* [43,44] in addition to *R. equi* [23]. PAM 2766^T^ assimilated a wide range of nitrogen sources, 41 out of 95 compounds tested in the PM3B plates (Fig. 3 and Table S2). As main differences with *R. equi*, PAM 2766^T^ was unable to grow on l-cysteine, l-lysine, l-citrulline, l-ornithine, cytosine, *ε*-amino-*N*-caproic acid and *δ*-amino-*N*-valeric acid. The relevant phenotypic characteristics of PAM 2766^T^ are summarized in Table 2 and below in the species description.

## Virulence

**Fig. 4.**
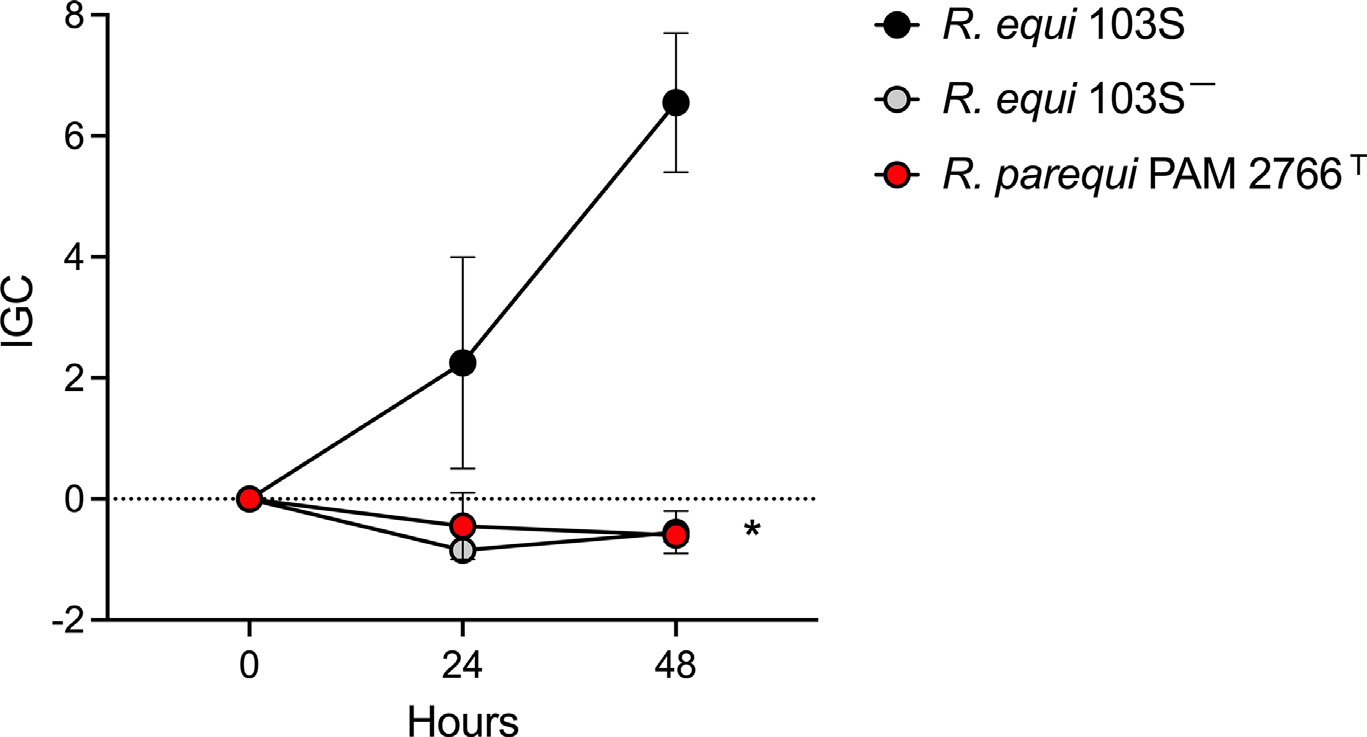
Macrophage infections. Intracellular proliferation phenotype of *R. parequi* PAM 2766^T^, virulent *R. equi* 103S (harbouring the pVAPA virulence plasmid that promotes intramacrophage replication [18,54, 55]) and non-virulent *R. equi* 103S^–^ (isogenic pVAPA-cured derivative unable to proliferate intracellularly [52]) in the J774A.1 murine macrophage-like cell line. J774A.1 cells (ATCC, <10 passages) were cultured in 24-well plates at 37 °C with 5% CO_2_ in RPMI supplemented with pyruvate and glutamine and 10% foetal bovine serum (Lonza) until confluence (approx. 2×10^5^ cells/well). Macrophage monolayers were infected at 10:1 multiplicity with washed bacteria from an exponential culture at 37 °C in TSB (OD_600_≈1.0). They were then centrifuged for 3 min at 172***g***, incubated for 60 min at 37 °C, washed three times with PBS to remove nonadherent bacteria and incubated in RPMI supplemented with 5 µg µl^−1^ vancomycin to prevent extracellular bacterial growth. After 1 h of incubation with vancomycin (*t* = 0) and at 24 and 48 h thereafter, macrophage monolayers were washed with PBS and lysed with 0.1% Triton X-100 for 3 min, and intracellular bacterial counts were determined by plating appropriate dilutions of the cell lysates onto TSA. As the intracellular bacterial population at a given time point depends on initial numbers, for data comparability between strains, bacterial intracellular kinetic data are expressed as a normalized intracellular growth coefficient (IGC)=(IB*_t_*_ = *n*_−IB*_t_*_ = 0_)/IB*_t_*_ = 0_, where IB*_t_*_ = *n*_ and IB*_t_*_ = 0_ are the intracellular bacterial numbers at a specific time point *t* = *n* and *t* = 0, respectively. Positive IGC indicates proliferation, and negative values reflect a decrease in the intracellular bacterial population. Bacterial counts per well at *t* = 0: 103S, 2.55±0.91×10^3^; 103S^–^, 1.75±0.17×10^3^; and *R. parequi* PAM 2766^T^, 1.67±0.18×10^3^. Means of two independent duplicate experiments±sem. The asterisk denotes significant differences at *t*=48 h with *R. equi* 103S (*P*≤0.001, two-way ANOVA).

Although PAM 2766^T^ was isolated from soil and we confirmed its genome lacked any of the plasmid virulence determinants of *R. equi* [45], this did not exclude that it might have pathogenic potential. To explore this, we tested PAM 2766^T^ in macrophage infection assays. The ability to survive and multiply within macrophages is the basis of the infectivity of *R. equi* and other related pathogenic actinomycetes [46,47]. Infection assays were performed in J774A.1 mouse macrophages cultured until confluence using a vancomycin protection assay as previously described [18,23] (see Fig. 4 legend for experimental details). Virulent *R. equi* 103S [23] and the non-virulent isogenic derivative 103S^–^ (obtained by the curation of the pVAPA virulence plasmid [48] required for intramacrophage survival [18,49, 50]) were used as positive and negative controls, respectively. *R. equi* 103S showed the expected behaviour, with significant intramacrophage proliferation during the infection time course. In contrast, PAM 2766^T^ intracellular numbers progressively declined, mirroring the behaviour of the pVAPA-cured, non-virulent *R. equi* 103S^–^ strain (Fig. 4). These data support the notion that PAM 2766^T^ is not primarily pathogenic and most likely represents an environmental saprotrophic species.

## Taxonomic conclusion

The clear-cut phylogenetic/genomic separation from the rest of the rhodococci and the identification of phenotypic markers allowing its differentiation from the closely related *R. equi* in our opinion justify the consideration of strain PAM 2766^T^ as a distinct *Rhodococcus* species. Owing to the remarkable morphological and biochemical similarities with *R. equi*, close phylogenetic relationship and similar source ecosystem, we propose for this novel species the name *Rhodococcus paraequi*. However, the final vowel of the prefix *para* has to be elided according to Appendix 9A(2) of the International Code of Nomenclature of Prokaryotes (ICNP) [51], giving *Rhodococcus parequi* sp. nov.

## Description of *Rhodococcus parequi* sp. nov.

*Rhodococcus parequi* (par.e’qui. Gr. prep. *para*, beside, alongside of, near, like; L. gen. n. *equi*, of a horse, specific epithet; N.L. gen. n. *parequi*, resembling *Rhodococcus equi*).

Members of this species are Gram-positive, strictly aerobic, non-spore-forming, non-motile, catalase-positive, oxidase-negative coccobacilli. *R. parequi* grows well in standard solid culture media such as TSA, BHI agar or LB agar. After 24–48-h incubation at 30 °C, it forms smooth, shiny, creamy to buff-coloured colonies that tend to coalesce over time. Colonies are morphologically very similar to those of *R. equi*. No haemolysis is observed on Columbia 5% sheep blood agar but gives a positive synergistic lytic (CAMP-like) reaction with sphingomyelinase C-producing bacteria such as *L. ivanovii* or *S. aureus* [13,52]. This ability is shared with *R. equi* and is the phenotypic expression of cholesterol oxidase production [12]. *R. parequi* also tests positive in a PCR targeting the *R. equi* cholesterol oxidase gene *choE* [14], considered up to now as a species-specific identification marker for *R. equi* [14,17]. The optimal growth temperature is between 30 and 37 °C. Tolerates NaCl concentrations up to 3% (w/v) and pH values from 5 to 9, with 7 as pH optimum. Tests positive for urea hydrolysis, nitrate reduction, acetoin production, tryptophane deaminase, *α*-glucosidase and alkaline phosphatase. It can be distinguished from *R. equi* [as tested with DSM 20307^T^ and 103S (PAM 1126)] by a rapid urease-positive reaction (at 24-h incubation), ability to grow at 5 °C; and inability to utilize Tween 20, 4-hydroxybenzoate and *β*-hydroxybutyrate as a carbon source and l-citrulline, l-ornithine, cytosine, *ε*-amino-*N*-caproic acid and *δ*-amino-*N*-valeric acid as a nitrogen source. The draft genome of strain PAM 2766^T^ is 5.3 Mbp in size with a digital G+C content of 68.98 mol%. The NCBI GenBank accession number for the genome assembly is JBDLNV000000000, and the accession number of the 16S rRNA gene sequence is PQ043279. Phylogenomically, *R. parequi* belongs to sublineage no. 2 of the *Rhodococcus* radiation (as defined in ref. [11]) together with *R. agglutinans*, *Rhodococcus defluvii*, *R. equi*, *R. soli*, *R. spongiicola*, *R. subtropicus* and *R. xishaensis*. Genomic similarity indices with its most closely related species *R. equi* are ANI=88.60 and AAI=92.35. *R. parequi* is avirulent in macrophage infection assays and is assumed to be a non-pathogenic environmental saprotroph.

The type strain is PAM 2766^T^ (CETC 30995^T^=NCTC 14987^T^) isolated from equine farm soil in Normandy, France.

## supplementary material

10.1099/ijsem.0.006679 Uncited Supplementary Material 1.

## Abbreviations

AAI: average aa identity
ANI: average nt identity
+ASC: ascertainment bias correction
BHI: brain heart infusion
CAMP: Christie–Atkins–Munch-Petersen test of synergistic haemolysis on sheep blood agar with sphingomyelinase C-producing indicator bacteria
CECT: Colección Española de Cultivos Tipo (Spanish Type Culture Collection)
COG: clusters of orthologous groups
dDDH: digital DNA–DNA hybridization
+F: empirical base frequencies
GGDC: Genome-to-Genome Distance Calculator
GTR: general time reversible with empirical base frequencies
LB: Luria–Bertani
LG: le gascuel general aa replacement matrix
LPSN: List of Prokaryotic names with Standing in Nomenclature
ML: maximum likelihood
NCTC: National Collection of Type Cultures
PAM: Patogénesis Microbiana isolate collection of Vázquez-Boland’s laboratory
+R: FreeRate model categories
TSA: tryptic soy agar
TSB: tryptic soy broth
WGS: whole-genome sequencing
