# Corrigendum: Rhodococcus parequi sp. nov., a new species isolated from equine farm soil closely related to the pathogen Rhodococcus equi

**DOI:** 10.1099/ijsem.0.007033

**Published:** 2026-01-13

**Authors:** José A. Vázquez-Boland, Jorge Val-Calvo, Fabien Duquesne, Francesca Decorosi, Carlo Viti, Sandrine Petry, Mariela Scortti

**Affiliations:** 1 Microbial Pathogenomics Laboratory and Rhodococcus equi Reference Resource, Edinburgh Medical School and Centre for Inflammation & Infection Research (CIR), University of Edinburgh, Edinburgh, UK; 2 ANSES, Laboratory for Animal Health, Physiopathology and Epidemiology of Equine Diseases Unit, Goustranville, France; 3 Department of Agriculture, Food, Environment and Forestry, University of Florence, Florence, Italy

In the originally published version of this article, an error was identified in the sequences of the universal primers 27F and 1492R reported in the first paragraph of the section ‘16S rRNA Gene Sequence’. The correct oligonucleotide primer sequences are as follows:

27F: 5′-AGAGTTTGATCCTGGCTCAG-3′ (20 nt)

1492R: 5′-CGGCTACCTTGTTACGACTT-3′ (20 nt)

The sequences of the oligonucleotide primers are based on those reported by Lane [1] except that our version of primer 1492R is two nucleotides shorter at the 5′ end (as often used by others). Additionally, both primers were optimised for *R. equi* and *R. parequi* by including the nucleotide C at the ‘M’ (C/A) position in 27F, and at the ‘Y’ (C/T) position in 1492R.

The authors apologise for any confusion or inconvenience caused.
