# Supplementary material for: Rhodococcus parequi sp. nov., a new species isolated from equine farm soil closely related to the pathogen Rhodococcus equi
Source: Int J Syst Evol Microbiol. 2025 Mar 10;75(3):006679. doi: 10.1099/ijsem.0.006679 (PMC11893733; doi:10.1099/ijsem.0.006679)
Supplement: Uncited Supplementary Material 1. [file ijsem-75-06679-s001.pdf]

SUPPLEMENTAL MATERIAL

Supplemental Figures

48h 30ºC | + 3d RT

R. parequi PAM 2766^T

R. equi DSM 20307^T

R. equi 103S

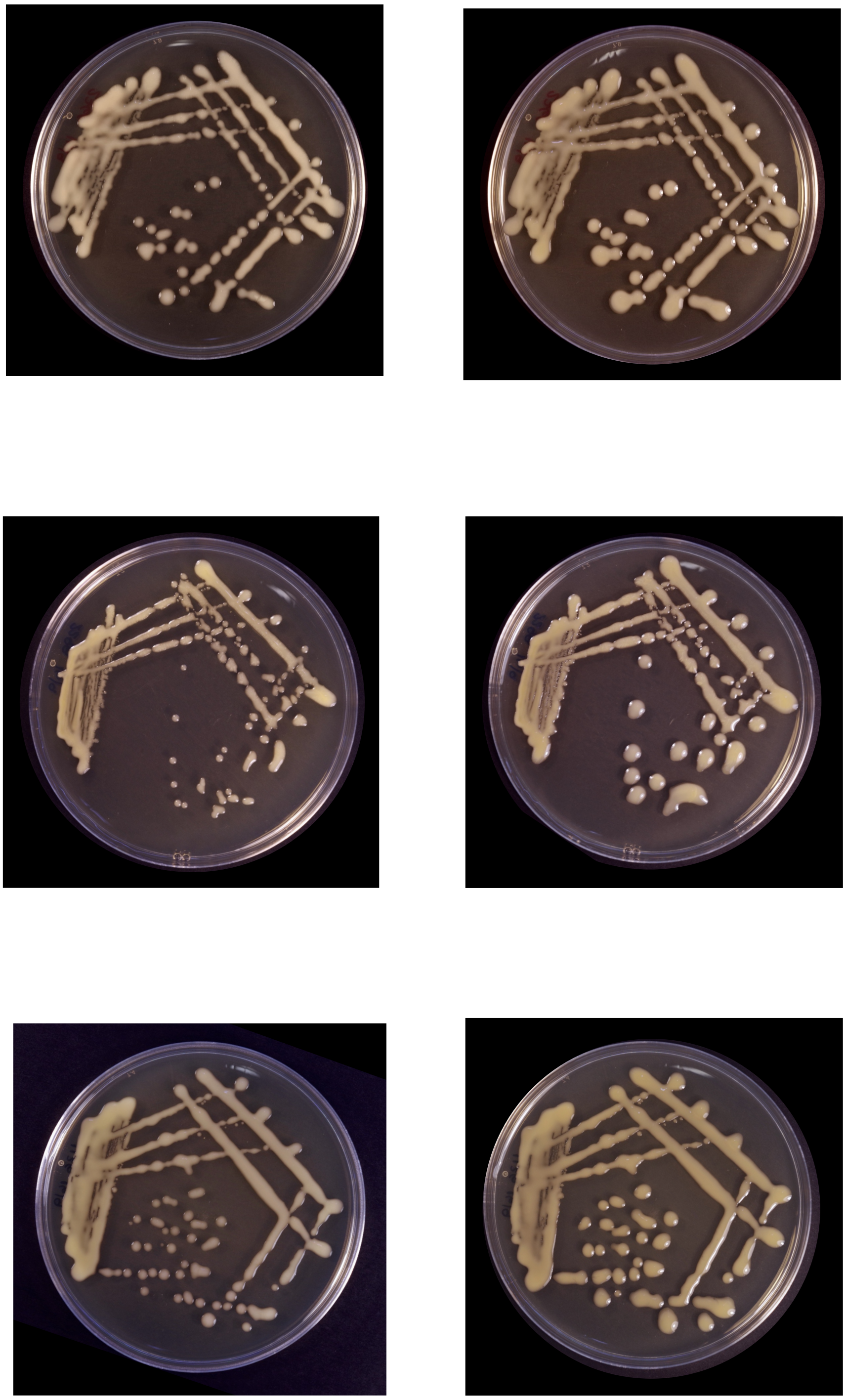

Fig. S1. Colony morphology of *R. parequi* PAM 2766 ^{T} compared to *R. equi* (type strain DSM 20307^T and reference genome strain 103S [PAM 1126], each belonging to one of the two main phylogenomic subdivisions of the species [29]). TSA plates were grown at 30 ºC for 48 h (left) and then left three additional days at room temperature.

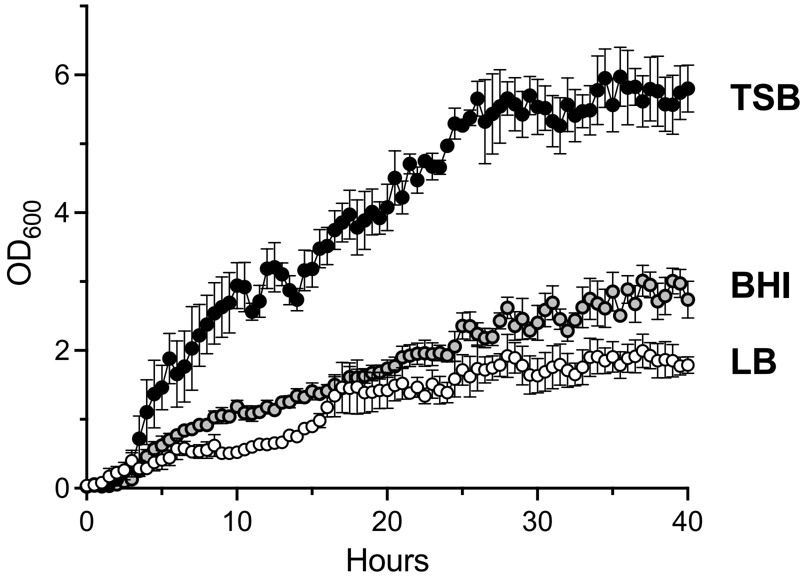

Fig. S2. Growth dynamics of PAM 2766T in different culture media: tryptic soy broth (TSB), brain-heart infusion (BHI), and Luria-Bertani broth (LB). Growth was monitored in 48-well plates (Costar) by measuring the OD_{600} every 30 min in an automated plate reader (Optima apparatus, BMG Labtech) during incubation at 30 ºC with 400 rpm shaking. Bacterial cells were obtained from an overnight culture in BHI incubated at 30 ºC, washed in PBS and resuspended in the appropriate medium to an optical density at 600nm (OD_{600}) ≈ 0.05. Wells were inoculated in duplicate using 400µl aliquots.

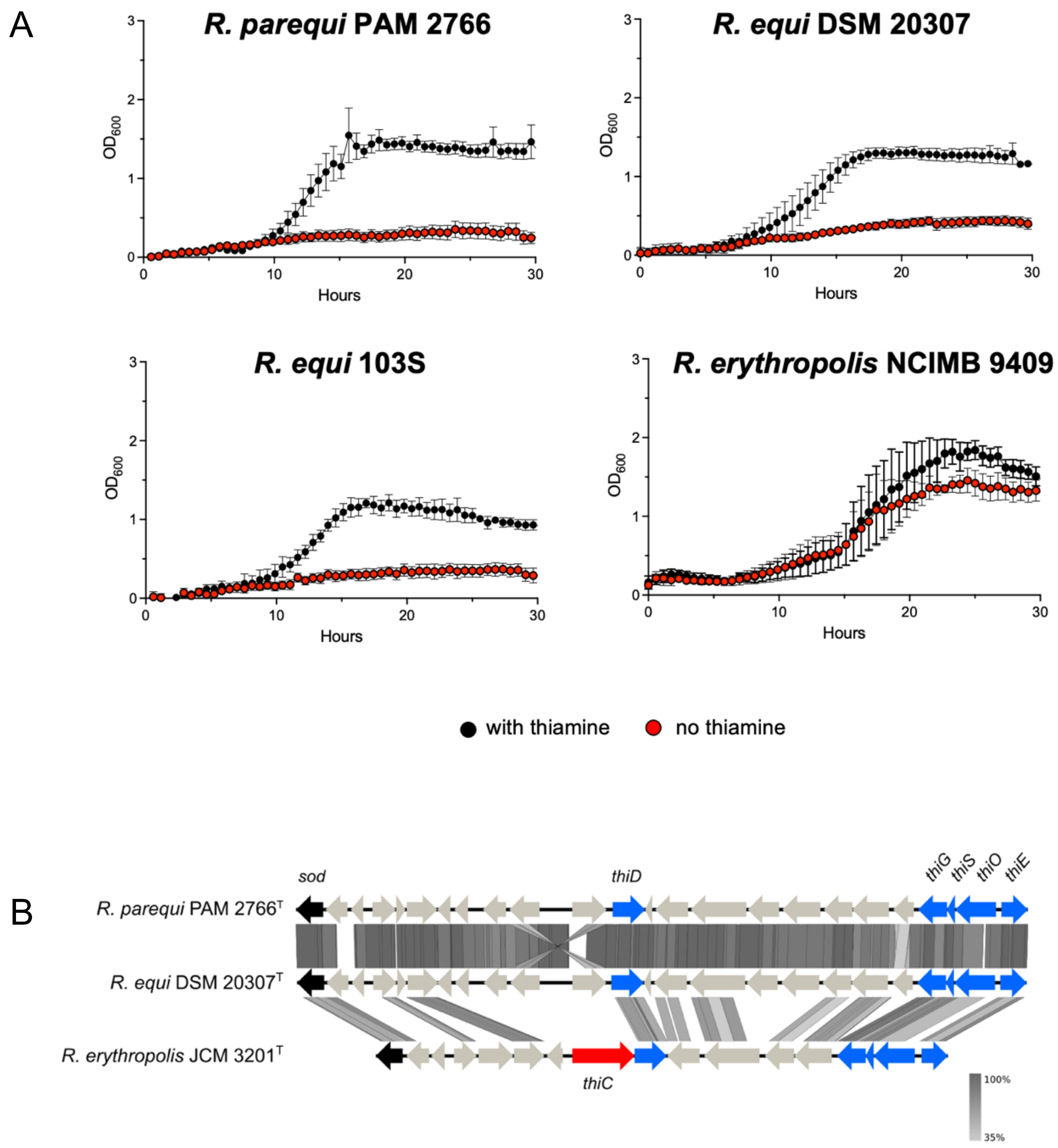

Fig. S3. Thiamine dependency of *R. parequi* PAM 2766^T. Determined in *R. equi* mineral medium [56] as modified in refs. [23, 29] (mReMM) supplemented with 20 mM lactate. (A) Growth curves with or without 0.1 mM thiamine supplementation of *R. parequi* PAM 2766^T, *R. equi* (two strains, one from each of the two main lineages of the species, DSM 20307^T and 103S) and a representative strain of *R. erythropolis*. Bacteria were grown overnight in TSB, collected by centrifugation, washed in PBS and resuspended in mReMM to $OD_{600} \approx 0.05$. Duplicate 400µl aliquots of each bacterial suspension were distributed in 48-well plates (Costar). Growth at 30ºC was monitored every 30 min in an automated plate reader (Omega, BMG Labtech) using double orbital shaking (400 rpm). Mean of at least three duplicate experiments ± SEM. Chemicals were purchased from Sigma. (B) TBlastX alignment of the chromosomal *thiCD-GSOE* thiamine biosynthesis locus of *R. equi* DSM 20307^T compared to that of *R. parequi* PAM 2766^T and *R. erythropolis* JCM 3201^T. Note that the genomic region is virtually identical in *R. equi* and *R. parequi*. Both species lack the *thiC* gene and are thiamine auxotrophs in contrast to *R. erythropolis* JCM 3201^T (as an example of a *Rhodococcus* sp. not requiring thiamine for efficient growth).

Table S1. Genome assemblies of type strains used in this study.

Species	Strain	NCBI assembly information					
		G+C content (%)	Size (Mbps)	No. of contigs/scaffolds	Completeness (%)	Contamination (%)	Accession number
Rhodococcoides fascians	NBRC 12155^T	64.5	5.8	36	99.5	1.46	GCF_001894785.1
Rhodococcus aetherivorans	DSM 44752^T	70.5	6.4	216	79.86	0.99	GCF_011058165.1
Rhodococcus agglutinans	CFHS 0262^T	69.0	5.4	22	99.5	1.12	GCF_004011865.1
Rhodococcus antarcticus	75^T	73.0	3.9	4	84.36	3.27	GCF_026153295.1
Rhodococcus coprophilus	NCTC 10994^T	67.0	4.6	1	96.8	1.04	GCF_900478115.1
Rhodococcus defluvii	Ca11^T	68.5	5.1	267	98.24	0.87	GCF_000738775.1
Rhodococcus electrodiphilus	LMG 29881^T	70.5	5.5	225	98.91	0	GCF_030509825.1
Rhodococcus equi	DMS 20307^T	69.0	5.2	37	98.33	0.99	GCF_002094305.1
Rhodococcus erythropolis	JCM 3201^T	62.5	6.7	3	94.82	4.28	GCF_003990875.1
Rhodococcus globerulus	NBRC 14531^T	61.5	6.7	30	99.19	0.78	GCF_001894805.1
Rhodococcus gordoniae	NCTC 13296^T	68.0	4.9	3	98.86	0.33	GCF_900455725.1
Rhodococcus indonesiensis	CSLK01-03^T	70.0	5.5	282	98.58	0	GCF_030360185.1
Rhodococcus jostii	DSM 44719^T	67.0	9.9	6	97.82	2.46	GCF_900105375.1
Rhodococcus koreensis	DSM 44498^T	67.5	10.3	9	99.35	4.95	GCF_900105905.1
Rhodococcus maanshanensis	DSM 44675^T	69.0	5.7	61	97.89	2.62	GCF_900109405.1
Rhodococcus marinonascens	NBRC 14363^T	64.5	4.9	156	95.52	1.1	GCF_001894885.1
Rhodococcus opacus	DSM 43205^T	67.0	9.0	38	99.5	3.59	GCF_910591545.1
Rhodococcus oryzae	NEAU-CX67^T	69.0	5.4	31	98.22	2.29	GCF_005049235.1
Rhodococcus oxybenzonivorans	S2-17^T	65.5	8.0	4	98.85	1.74	GCF_003130705.1
Rhodococcus phenolicus	DSM 44812^T	68.5	6.3	232	97.45	2.16	GCF_001646785.1
Rhodococcus pseudokoreensis	R79^T	67.5	9.9	6	99.35	3.24	GCF_017068395.1
Rhodococcus pyridinivorans	DSM 44555^T	68.0	5.3	3	96.81	2.53	GCF_900105195.1
Rhodococcus rhodnii	ATCC 35071^T	69.5	4.5	4	92.35	1.03	GCF_008011915.1
Rhodococcus rhodochrous	NCTC 10210^T	68.0	5.3	1	99.44	0.99	GCF_900187265.1
Rhodococcus ruber	NBRC 15591^T	70.5	5.3	56	99.27	0.37	GCF_001894945.1
Rhodococcus soli	DSM 46662^T [a]	68.8	5.4	20	98.71	0.68	JBDLNU000000000
Rhodococcus spelaei	C9-5^T	69.0	4.8	18	97.11	1.21	GCF_006704125.1
Rhodococcus spongiicola	LHW 50502^T	66.5	4.0	29	96.65	1.3	GCF_004011835.1

[a] The genome sequence of *R. soli* DSM 46662^T was unavailable at the time of the study and was determined *de novo*.

Table S1. (cont.)

Species	Strain	NCBI assembly information					
		G+C content (%)	Size (Mbps)	number of contigs/scaffolds	Completeness (%)	Contamination (%)	Accession number
Rhodococcus subtropicus	C9-28^T	69	4.4	67	98.2	0.65	GCF_005434945.1
Rhodococcus triatomae	DSM 44892^T	68.5	4.8	1	97.77	1.21	GCF_014217785.1
Rhodococcus tukisamuensis	JCM 11308^T	70.0	5.5	40	97.62	1.66	GCF_900101735.1
Rhodococcus wratislaviensis	NCTC 13229^T	67.5	7.8	29	99.19	1.24	GCF_900455735.1
Rhodococcus xishaensis	LHW51113^T	66.5	3.7	22	96.71	0.8	GCF_004011825.1
Rhodococcus yananensis	FBM 22-1^T	68.5	4.2	178	94.65	0.91	GCF_020515525.1
Rhodococcus zopfii	NBRC 100606^T	68	6.3	146	98.61	3.24	GCF_001895025.1

Table S2. Phenotype MicroArray data.

Plate	Well	Chemical	Mode of action	Maximmun growth rate					
				PAM 2766		*R. equi* DSM 20307^T		*R. equi* 103S PAM 1126	
				Mean	SEM	Mean	SEM	Mean	SEM
PM1	A01	Negative Control	C-Source, negative contro	0.1650	0.0550	0.2350	0.0550	0.1700	0.0100
PM1	A02	L-Arabinose	C-Source, carbohydrate	1.3200	0.3800	1.3400	0.2400	0.9600	0.2600
PM1	A03	N-Acetyl-D-Glucosamine	C-Source, carbohydrate	0.2000	0.0800	0.2250	0.0050	0.3300	0.1600
PM1	A04	D-Saccharic acid	C-Source, carboxylic acid	0.1100	0.0700	0.1950	0.0650	0.2050	0.0250
PM1	A05	Succinic acid	C-Source, carboxylic acid	3.3000	1.1900	6.0750	0.3750	4.8950	4.7050
PM1	A06	D-Galactose	C-Source, carbohydrate	0.1350	0.0050	0.2350	0.0250	0.1950	0.0350
PM1	A07	L-Aspartic acid	C-Source, amino acid	0.3050	0.2750	1.1650	1.0650	0.6750	0.5850
PM1	A08	L-Proline	C-Source, amino acid	0.2050	0.1550	0.4450	0.3350	0.5250	0.4550
PM1	A09	D-Alanine	C-Source, amino acid	0.3350	0.3150	0.2900	0.1600	0.1300	0.0900
PM1	A10	D-Trehalose	C-Source, carbohydrate	0.1400	0.0400	0.4150	0.1250	0.1250	0.0050
PM1	A11	D-Mannose	C-Source, carbohydrate	0.1250	0.0650	0.3300	0.0000	0.1300	0.0300
PM1	A12	Dulcitol	C-Source, carbohydrate	0.0550	0.0650	0.1350	0.0150	0.5000	0.4100
PM1	B01	D-Serine	C-Source, amino acid	0.2200	0.0400	0.1750	0.0250	0.1800	0.0200
PM1	B02	D-Sorbitol	C-Source, carbohydrate	0.1200	0.0500	0.2050	0.0450	0.5800	0.3800
PM1	B03	Glycerol	C-Source, carbohydrate	0.0550	0.0150	0.2700	0.0300	0.3500	0.0200
PM1	B04	L-Fucose	C-Source, carbohydrate	0.1550	0.0050	0.4000	0.1300	0.2500	0.0600
PM1	B05	D-Glucuronic acid	C-Source, carboxylic acid	0.2150	0.1550	0.2950	0.1450	0.1750	0.0450
PM1	B06	D-Gluconic acid	C-Source, carboxylic acid	0.1500	0.0900	0.2550	0.0450	0.1950	0.0750
PM1	B07	D,L-a-Glycerol Phosphate	C-Source, carbohydrate	0.1000	0.0300	0.1900	0.0300	0.1600	0.0500
PM1	B08	D-Xylose	C-Source, carbohydrate	2.5450	0.8550	2.1900	0.6700	2.7350	0.0450
PM1	B09	L-Lactic acid	C-Source, carboxylic acid	8.0100	0.6900	19.2250	2.0350	19.7850	0.3450
PM1	B10	Formic acid	C-Source, carboxylic acid	0.0900	0.0600	0.1750	0.0650	0.0850	0.0650
PM1	B11	D-Mannitol	C-Source, carbohydrate	0.1000	0.0600	0.1700	0.0500	0.1250	0.0650
PM1	B12	L-Glutamic acid	C-Source, amino acid	0.0150	0.0150	0.9200	0.2400	0.1550	0.0150
PM1	C01	D-Glucose-6-Phosphate	C-Source, carbohydrate	0.1400	0.0600	0.2150	0.0750	0.1600	0.0400
PM1	C02	D-Galactonic acid-g-Lactone	C-Source, carboxylic acid	0.0950	0.0150	0.1000	0.0100	0.0600	0.0000
PM1	C03	D,L-Malic acid	C-Source, carboxylic acid	5.1850	1.6850	9.7950	0.4550	6.7350	0.6450
PM1	C04	D-Ribose	C-Source, carbohydrate	8.5050	2.9750	14.7300	4.2700	5.7600	0.8700
PM1	C05	Tween 20	C-Source, fatty acid	0.7400	0.2100	16.4200	0.3000	14.6500	1.3500
PM1	C06	L-Rhamnose	C-Source, carbohydrate	0.4850	0.3250	0.4350	0.1850	0.2300	0.0200
PM1	C07	D-Fructose	C-Source, carbohydrate	0.1300	0.0100	0.3450	0.1550	0.3400	0.1700
PM1	C08	Acetic acid	C-Source, carboxylic acid	7.9200	0.0000	10.5500	0.1800	21.7300	0.2100
PM1	C09	a-D-Glucose	C-Source, carbohydrate	0.0800	0.0300	2.7850	0.3750	0.1700	0.0400
PM1	C10	Maltose	C-Source, carbohydrate	0.0650	0.0150	0.2050	0.0350	0.1300	0.0100
PM1	C11	D-Melibiose	C-Source, carbohydrate	0.0850	0.0150	0.2900	0.1500	0.1000	0.0100
PM1	C12	Thymidine	C-Source, carbohydrate	0.0950	0.0850	0.1600	0.0300	0.1150	0.0450
PM1	D01	L-Asparagine	C-Source, amino acid	0.1150	0.0350	0.1350	0.0050	0.1400	0.0100
PM1	D02	D-Aspartic acid	C-Source, amino acid	0.0750	0.0150	0.1350	0.0350	0.1400	0.0100
PM1	D03	D-Glucosaminic acid	C-Source, carboxylic acid	0.0550	0.0050	0.2650	0.1150	0.1350	0.0050
PM1	D04	1,2-Propanediol	C-Source, alcohol	0.0650	0.0450	0.4600	0.1100	0.3650	0.0450
PM1	D05	Tween 40	C-Source, fatty acid	21.2050	0.2250	17.2950	2.1050	30.7750	2.1250
PM1	D06	a-Ketoglutaric acid	C-Source, carboxylic acid	0.8200	0.1400	0.6050	0.1750	0.4600	0.1300
PM1	D07	a-Ketobutyric acid	C-Source, carboxylic acid	0.6150	0.0050	0.6600	0.0300	0.8550	0.0450
PM1	D08	a-Methyl-D-Galactoside	C-Source, carbohydrate	0.1350	0.0450	0.2950	0.1850	0.1500	0.0300
PM1	D09	a-D-Lactose	C-Source, carbohydrate	0.0850	0.0350	0.2100	0.0300	0.1200	0.0300
PM1	D10	Lactulose	C-Source, carbohydrate	0.0800	0.0400	0.2200	0.0400	0.1100	0.0300
PM1	D11	Sucrose	C-Source, carbohydrate	0.1200	0.0600	0.2650	0.0350	0.1700	0.0400
PM1	D12	Uridine	C-Source, carbohydrate	0.0650	0.0250	0.1200	0.0200	0.1000	0.0200
PM1	E01	L-Glutamine	C-Source, amino acid	0.2100	0.0800	0.2600	0.0200	0.1600	0.0300
PM1	E02	m-Tartaric acid	C-Source, carboxylic acid	0.0800	0.0300	0.1350	0.0550	0.1250	0.0150
PM1	E03	D-Glucose-1-Phosphate	C-Source, carbohydrate	0.0900	0.0400	0.1500	0.0200	0.1150	0.0150
PM1	E04	D-Fructose-6-Phosphate	C-Source, carbohydrate	0.1550	0.0050	0.2700	0.0100	0.2050	0.0050
PM1	E05	Tween 80	C-Source, fatty acid	21.7700	0.4200	21.4750	0.4750	26.9400	0.3200
PM1	E06	a-Hydroxyglutaric acid-g-La	C-Source, carboxylic acid	0.0700	0.0100	0.1700	0.0400	0.1300	0.0100
PM1	E07	a-Hydroxybutyric acid	C-Source, carboxylic acid	0.3550	0.0450	0.2050	0.0450	1.2600	0.0100
PM1	E08	b-Methyl-D-Glucoside	C-Source, carbohydrate	0.2850	0.2450	0.3000	0.1600	0.5400	0.3900
PM1	E09	Adonitol	C-Source, carbohydrate	0.0750	0.0250	0.2000	0.0200	0.1300	0.0100
PM1	E10	Maltotriose	C-Source, carbohydrate	0.0200	0.0400	0.7500	0.0400	0.2100	0.0200
PM1	E11	2`-Deoxyadenosine	C-Source, carbohydrate	0.0500	0.0300	-0.0500	0.0300	0.0500	0.0300
PM1	E12	Adenosine	C-Source, carbohydrate	0.0650	0.0850	0.0450	0.0350	0.1300	0.0600
PM1	F01	Gly-Asp	C-Source, amino acid	0.0800	0.0300	0.2250	0.0550	0.1300	0.0100
PM1	F02	Citric acid	C-Source, carboxylic acid	0.0700	0.0200	0.1000	0.0000	0.1100	0.0200
PM1	F03	m-Inositol	C-Source, carbohydrate	0.0650	0.0150	0.1800	0.0200	0.1400	0.0100
PM1	F04	D-Threonine	C-Source, amino acid	0.0500	0.0000	0.1550	0.0150	0.1300	0.0300
PM1	F05	Fumaric acid	C-Source, carboxylic acid	5.8000	0.6400	7.2900	0.8100	5.1150	0.6350
PM1	F06	Bromosuccinic acid	C-Source, carboxylic acid	4.5050	0.1650	6.8000	0.2500	6.0900	0.4900
PM1	F07	Propionic acid	C-Source, carboxylic acid	6.0350	0.6050	9.6400	0.5000	16.2150	2.1150
PM1	F08	Mucic acid	C-Source, carboxylic acid	0.0500	0.0000	0.1350	0.0050	0.1700	0.0300
PM1	F09	Glycolic acid	C-Source, carboxylic acid	0.0350	0.0150	0.0700	0.0200	0.0400	0.0200
PM1	F10	Glyoxylic acid	C-Source, carboxylic acid	0.2250	0.0050	0.2400	0.0400	0.2450	0.0250
PM1	F11	D-Cellobiose	C-Source, carbohydrate	0.0600	0.0100	0.1850	0.0050	0.1400	0.0000
PM1	F12	Inosine	C-Source, carbohydrate	0.0200	0.0100	0.1400	0.0200	0.1250	0.0150
PM1	G01	Gly-Glu	C-Source, amino acid	0.1250	0.0350	0.1750	0.0750	0.1200	0.0300
PM1	G02	Tricarballylic acid	C-Source, carboxylic acid	0.1050	0.0150	0.1950	0.0250	0.1100	0.0500
PM1	G03	L-Serine	C-Source, amino acid	0.0650	0.0150	0.0750	0.0250	0.0600	0.0300
PM1	G04	L-Threonine	C-Source, amino acid	0.0650	0.0050	0.0950	0.0050	0.1250	0.0050
PM1	G05	L-Alanine	C-Source, amino acid	0.0250	0.0250	0.0550	0.0150	0.0650	0.0050
PM1	G06	Ala-Gly	C-Source, amino acid	0.0750	0.0350	0.1000	0.0500	0.3400	0.2000
PM1	G07	Acetoacetic acid	C-Source, carboxylic acid	1.6750	1.6050	1.1550	0.0050	1.3700	0.2300
PM1	G08	N-Acetyl-D-Mannosamine	C-Source, carbohydrate	0.0700	0.0200	0.1650	0.0250	0.1200	0.0400
PM1	G09	Mono-Methylsuccinate	C-Source, carboxylic acid	3.6650	0.1150	4.8800	0.1700	2.5500	2.3100
PM1	G10	Methylpyruvate	C-Source, ester	3.2650	0.0850	15.7250	0.6350	7.5700	3.1900
PM1	G11	D-Malic acid	C-Source, carboxylic acid	0.2050	0.0350	0.1850	0.0250	0.2350	0.0350
PM1	G12	L-Malic acid	C-Source, carboxylic acid	6.2450	0.2450	9.6000	0.0700	8.4300	0.1300
PM1	H01	Gly-Pro	C-Source, amino acid	0.0450	0.0050	0.1150	0.0550	0.0300	0.0100
PM1	H02	p-Hydroxyphenyl Acetic acic	C-Source, carboxylic acid	0.0900	0.0100	0.1150	0.0450	0.0900	0.0100
PM1	H03	m-Hydroxyphenyl Acetic aci	C-Source, carboxylic acid	6.1000	0.3800	9.1100	0.2000	10.8300	0.2900
PM1	H04	Tyramine	C-Source, amine	0.0500	0.0300	0.0400	0.0300	0.0500	0.0000
PM1	H05	D-Psicose	C-Source, carbohydrate	0.1700	0.0100	0.1750	0.0050	0.1750	0.0350
PM1	H06	L-Lyxose	C-Source, carbohydrate	17.4900	3.5000	17.9650	2.1450	13.2100	0.6500
PM1	H07	Glucuronamide	C-Source, amide	0.2200	0.0000	0.4350	0.1150	0.2350	0.0150
PM1	H08	Pyruvic acid	C-Source, carboxylic acid	2.5850	0.6950	16.9650	0.4950	9.6050	3.3950
PM1	H09	L-Galactonic acid-g-Lactone	C-Source, carboxylic acid	0.0850	0.0350	0.1650	0.0750	0.1950	0.0150
PM1	H10	D-Galacturonic acid	C-Source, carboxylic acid	0.0700	0.0300	0.1550	0.0350	0.1450	0.0350
PM1	H11	Phenylethylamine	C-Source, amine	-0.0150	0.0450	0.1350	0.0050	0.0700	0.0000
PM1	H12	2-Aminoethanol	C-Source, alcohol	0.0500	0.0100	0.0800	0.0300	0.0750	0.0350

Plate	Well	Chemical	Mode of action	Maximmun growth rate					
				PAM 2766		*R. equi* DSM 20307[T]		*R. equi* 103S PAM 1126	
				Mean	SEM	Mean	SEM	Mean	SEM
PM2A	A01	Negative Control	C-Source, negative contro	0.1200	0.0300	0.1900	0.0300	0.2000	0.0100
PM2A	A02	Chondroitin Sulfate C	C-Source, polymer	0.1250	0.0550	0.2100	0.1300	0.1900	0.0000
PM2A	A03	a-Cyclodextrin	C-Source, polymer	0.1850	0.0350	0.7450	0.2850	0.9650	0.2350
PM2A	A04	b-Cyclodextrin	C-Source, polymer	0.1150	0.0350	0.4450	0.1450	0.2600	0.0300
PM2A	A05	g-Cyclodextrin	C-Source, polymer	0.2950	0.0550	0.7100	0.1100	0.8950	0.0550
PM2A	A06	Dextrin	C-Source, polymer	0.2950	0.1150	0.7450	0.0950	0.2550	0.0150
PM2A	A07	Gelatin	C-Source, polymer	0.1150	0.0450	0.7050	0.2150	0.1450	0.0450
PM2A	A08	Glycogen	C-Source, polymer	0.0150	0.0350	0.4150	0.2550	0.1850	0.0550
PM2A	A09	Inulin	C-Source, polymer	0.0550	0.0350	0.4550	0.3750	0.1700	0.1000
PM2A	A10	Laminarin	C-Source, polymer	0.0900	0.0000	0.6550	0.2250	0.1350	0.0250
PM2A	A11	Mannan	C-Source, polymer	0.0600	0.0000	0.2650	0.1350	0.1800	0.0200
PM2A	A12	Pectin	C-Source, polymer	0.0600	0.0000	0.4200	0.0600	0.1300	0.0100
PM2A	B01	N-Acetyl-D-Galactosamine	C-Source, carbohydrate	0.1000	0.0200	0.2800	0.0600	0.1850	0.0650
PM2A	B02	N-Acetyl-Neuraminic acid	C-Source, carboxylic acid	0.0250	0.0150	0.1250	0.0450	0.0600	0.0000
PM2A	B03	b-D-Allose	C-Source, carbohydrate	0.0950	0.0050	0.1450	0.0150	0.2000	0.0300
PM2A	B04	Amygdalin	C-Source, carbohydrate	0.0300	0.0100	0.2700	0.1200	0.1250	0.0850
PM2A	B05	D-Arabinose	C-Source, carbohydrate	0.6100	0.1600	1.3200	0.1700	1.2150	0.0150
PM2A	B06	D-Arabitol	C-Source, carbohydrate	0.0550	0.0350	0.2950	0.1750	0.2450	0.1350
PM2A	B07	L-Arabitol	C-Source, carbohydrate	0.0100	0.0200	0.2250	0.0850	0.1750	0.0750
PM2A	B08	Arbutin	C-Source, carbohydrate	0.0300	0.0100	0.2500	0.1500	0.1300	0.0500
PM2A	B09	2-Deoxy-D-Ribose	C-Source, carbohydrate	8.6250	0.2050	11.9850	2.6350	9.0300	0.1400
PM2A	B10	i-Erythritol	C-Source, carbohydrate	0.0150	0.0150	0.2500	0.1300	0.1400	0.0500
PM2A	B11	D-Fucose	C-Source, carbohydrate	0.0550	0.0250	0.2800	0.1800	0.1450	0.0550
PM2A	B12	3-0-b-D-Galactopyranosyl-D	C-Source, carbohydrate	0.0350	0.0050	0.1250	0.1250	0.0450	0.0050
PM2A	C01	Gentiobiose	C-Source, carbohydrate	0.0900	0.0200	0.3250	0.0550	0.2200	0.0300
PM2A	C02	L-Glucose	C-Source, carbohydrate	0.0300	0.0100	0.2800	0.1500	0.1150	0.0450
PM2A	C03	D-Lactitol	C-Source, carbohydrate	0.0100	0.0000	0.1600	0.0100	0.0950	0.0650
PM2A	C04	D-Melezitose	C-Source, carbohydrate	0.0550	0.0050	0.5500	0.3200	0.2950	0.0950
PM2A	C05	Maltitol	C-Source, carbohydrate	0.0100	0.0100	0.3050	0.1550	0.1400	0.0100
PM2A	C06	a-Methyl-D-Glucoside	C-Source, carbohydrate	0.0400	0.0000	0.2750	0.1250	0.1150	0.0250
PM2A	C07	b-Methyl-D-Galactoside	C-Source, carbohydrate	0.0250	0.0050	0.2650	0.1450	0.1250	0.0250
PM2A	C08	3-Methylglucose	C-Source, carbohydrate	0.0350	0.0050	0.1450	0.1250	0.1400	0.0300
PM2A	C09	b-Methyl-D-Glucuronic acid	C-Source, carboxylic acid	0.0450	0.0150	0.1000	0.0100	0.0750	0.0150
PM2A	C10	a-Methyl-D-Mannoside	C-Source, carbohydrate	0.0350	0.0150	0.1850	0.0650	0.1000	0.0100
PM2A	C11	b-Methyl-D-Xyloside	C-Source, carbohydrate	0.0250	0.0150	0.1900	0.1500	0.0600	0.0200
PM2A	C12	Palatinose	C-Source, carbohydrate	0.1700	0.0000	0.3250	0.1050	0.2350	0.0050
PM2A	D01	D-Raffinose	C-Source, carbohydrate	0.1000	0.0100	0.1700	0.0100	0.2200	0.0200
PM2A	D02	Salicin	C-Source, carbohydrate	0.0150	0.0150	0.1500	0.0500	0.0450	0.0450
PM2A	D03	Sedoheptulosan	C-Source, carbohydrate	0.0600	0.0500	0.1600	0.0100	0.1050	0.0050
PM2A	D04	L-Sorbose	C-Source, carbohydrate	0.0750	0.0150	0.1200	0.0100	0.4100	0.3000
PM2A	D05	Stachyose	C-Source, carbohydrate	0.0400	0.0000	0.2000	0.0700	0.1800	0.0500
PM2A	D06	D-Tagatose	C-Source, carbohydrate	0.2150	0.0150	0.3350	0.1250	0.2700	0.0600
PM2A	D07	Turanose	C-Source, carbohydrate	0.0650	0.0050	0.5350	0.1450	0.3250	0.0350
PM2A	D08	Xylitol	C-Source, carbohydrate	0.0200	0.0100	0.1550	0.0450	0.1300	0.0100
PM2A	D09	N-Acetyl-D-Glucosaminitol	C-Source, carbohydrate	0.1200	0.0500	0.3100	0.1300	0.0950	0.0450
PM2A	D10	g-Amino-N-Butyric acid	C-Source, carboxylic acid	0.0300	0.0000	0.2200	0.1600	0.0650	0.0050
PM2A	D11	d-Amino Valeric acid	C-Source, carboxylic acid	0.0150	0.0050	0.1750	0.1250	0.0700	0.0400
PM2A	D12	Butyric acid	C-Source, carboxylic acid	7.3450	0.1750	17.6150	0.1350	17.5300	1.3800
PM2A	E01	Capric acid	C-Source, carboxylic acid	0.0850	0.0350	0.0750	0.0150	0.0500	0.0400
PM2A	E02	Caproic acid	C-Source, carboxylic acid	2.2750	0.1950	18.7750	0.6750	15.6150	2.8350
PM2A	E03	Citraconic acid	C-Source, carboxylic acid	0.0400	0.0300	0.1150	0.0350	0.1050	0.0450
PM2A	E04	D,L-Citramalic acid	C-Source, carboxylic acid	0.0350	0.0050	0.1250	0.0150	0.1400	0.0300
PM2A	E05	D-Glucosamine	C-Source, carbohydrate	0.8050	0.0050	0.8100	0.0100	0.7500	0.0300
PM2A	E06	2-Hydroxybenzoic acid	C-Source, carboxylic acid	0.0050	0.0150	0.0100	0.0400	0.0850	0.0350
PM2A	E07	4-Hydroxybenzoic acid	C-Source, carboxylic acid	0.0450	0.0150	6.9500	0.4100	6.8750	1.1850
PM2A	E08	b-Hydroxybutyric acid	C-Source, carboxylic acid	0.5050	0.0150	5.3450	0.0650	5.2900	0.3000
PM2A	E09	g-Hydroxybutyric acid	C-Source, carboxylic acid	0.0150	0.0050	0.0950	0.0350	-0.0050	0.0150
PM2A	E10	a-Keto-Valeric acid	C-Source, carboxylic acid	1.3400	0.0100	3.0900	0.6200	1.8100	0.6900
PM2A	E11	Itaconic acid	C-Source, carboxylic acid	0.0050	0.0150	0.2050	0.1450	0.0500	0.0200
PM2A	E12	5-Keto-D-Gluconic acid	C-Source, carboxylic acid	1.0700	0.1700	1.2550	0.0350	1.3500	0.1600
PM2A	F01	D-Lactic acid Methyl Ester	C-Source, ester	1.0850	0.2650	5.7400	0.8800	0.1850	0.0950
PM2A	F02	Malonic acid	C-Source, carboxylic acid	0.0500	0.0300	0.1350	0.0050	0.1050	0.0550
PM2A	F03	Melibionic acid	C-Source, carbohydrate	0.0400	0.0400	0.0550	0.1050	0.1250	0.0250
PM2A	F04	Oxalic acid	C-Source, carboxylic acid	0.0300	0.0000	0.1100	0.0300	0.1300	0.0000
PM2A	F05	Oxalomalic acid	C-Source, carboxylic acid	0.9200	0.2500	1.2450	0.0550	1.3050	0.1050
PM2A	F06	Quinic acid	C-Source, carboxylic acid	0.0700	0.0200	0.2550	0.1250	0.1550	0.0050
PM2A	F07	D-Ribono-1,4-Lactone	C-Source, carboxylic acid	0.0800	0.0100	0.0900	0.0100	0.1050	0.0050
PM2A	F08	Sebacic acid	C-Source, carboxylic acid	3.3450	0.3050	0.4950	0.0650	0.5100	0.0300
PM2A	F09	Sorbic acid	C-Source, carboxylic acid	2.2300	0.2300	3.1400	1.0500	3.4700	1.0100
PM2A	F10	Succinamic acid	C-Source, carboxylic acid	0.2000	0.0800	2.7000	0.2400	0.1250	0.0050
PM2A	F11	D-Tartaric acid	C-Source, carboxylic acid	0.0450	0.0150	0.0800	0.0200	0.0950	0.0250
PM2A	F12	L-Tartaric acid	C-Source, carboxylic acid	0.0000	0.0300	0.1400	0.0100	0.0950	0.0050
PM2A	G01	Acetamide	C-Source, amide	0.0850	0.0450	0.1350	0.0750	6.7900	6.6500
PM2A	G02	L-Alaninamide	C-Source, amide	0.0150	0.0250	0.0450	0.0250	0.0700	0.0300
PM2A	G03	N-Acetyl-L-Glutamic acid	C-Source, amino acid	0.0250	0.0050	0.1200	0.0500	0.1150	0.0150
PM2A	G04	L-Arginine	C-Source, amino acid	0.0150	0.0050	0.0850	0.0050	0.0850	0.0050
PM2A	G05	Glycine	C-Source, amino acid	0.0350	0.0150	0.0550	0.0050	0.0800	0.0000
PM2A	G06	L-Histidine	C-Source, amino acid	4.7400	0.1200	1.7250	1.4650	4.0450	0.2750
PM2A	G07	L-Homoserine	C-Source, amino acid	0.0200	0.0000	0.1000	0.0300	0.0950	0.0050
PM2A	G08	Hydroxy-L-Proline	C-Source, amino acid	0.0450	0.0350	0.1300	0.0100	0.1200	0.0100
PM2A	G09	L-Isoleucine	C-Source, amino acid	1.5050	1.4750	0.1400	0.0200	0.1700	0.0800
PM2A	G10	L-Leucine	C-Source, amino acid	3.1350	3.0150	0.2000	0.0300	0.0950	0.0050
PM2A	G11	L-Lysine	C-Source, amino acid	0.0050	0.0050	0.1550	0.0950	0.0250	0.0050
PM2A	G12	L-Methionine	C-Source, amino acid	0.0450	0.0550	0.0950	0.0150	0.1350	0.0950
PM2A	H01	L-Ornithine	C-Source, amino acid	0.0550	0.0450	0.0950	0.0050	0.0800	0.0500
PM2A	H02	L-Phenylalanine	C-Source, amino acid	0.0700	0.0100	0.0700	0.0100	0.0800	0.0500
PM2A	H03	L-Pyroglutamic acid	C-Source, amino acid	0.0150	0.0250	0.1650	0.0250	0.1150	0.0150
PM2A	H04	L-Valine	C-Source, amino acid	0.0650	0.0150	0.0900	0.0400	0.1150	0.0250
PM2A	H05	D,L-Carnitine	C-Source, carboxylic acid	0.0550	0.0450	0.0950	0.0250	0.0850	0.0050
PM2A	H06	sec-Butylamine	C-Source, amine	0.0450	0.0350	0.1050	0.0150	0.0850	0.0150
PM2A	H07	D,L-Octopamine	C-Source, amine	0.0550	0.0350	0.1400	0.0000	0.1100	0.0000
PM2A	H08	Putrescine	C-Source, amine	0.0550	0.0050	0.0850	0.0150	0.0600	0.0100
PM2A	H09	Dihydroxyacetone	C-Source, alcohol	3.8400	0.1000	9.0450	5.3250	3.6750	0.0150
PM2A	H10	2,3-Butanediol	C-Source, alcohol	0.0300	0.0100	0.1100	0.0100	0.0750	0.0150
PM2A	H11	2,3-Butanone	C-Source, alcohol	0.5300	0.0600	0.5400	0.0000	0.9150	0.1250
PM2A	H12	3-Hydroxy-2-butanone	C-Source, alcohol	0.0300	0.0400	0.0600	0.0100	0.0300	0.0100

Plate	Well	Chemical	Mode of action	Maximmun growth rate					
				PAM 2766		*R. equi* DSM 20307[T]		*R. equi* 103S PAM 1126	
				Mean	SEM	Mean	SEM	Mean	SEM
PM3B	A01	Negative Control	N-Source, Negative contro	1.1900	0.0141	0.9050	0.1485	1.4350	0.0495
PM3B	A02	Ammonia	N-Source, inorganic	9.4900	0.0707	12.4950	1.7466	21.5500	2.0365
PM3B	A03	Nitrite	N-Source, inorganic	6.2150	0.2333	8.0900	0.4525	13.7350	0.0071
PM3B	A04	Nitrate	N-Source, inorganic	5.8650	0.2192	6.9650	0.0778	13.3800	0.0849
PM3B	A05	Urea	N-Source, other	7.7400	0.1980	8.1550	0.4031	16.2800	2.1496
PM3B	A06	Biuret	N-Source, other	1.7950	0.1768	0.6600	0.0141	1.8500	0.0424
PM3B	A07	L-Alanine	N-Source, amino acid	1.9450	0.0919	2.1700	0.0424	2.2350	0.0636
PM3B	A08	L-Arginine	N-Source, amino acid	1.4600	0.0707	0.7050	0.1344	1.5550	0.2475
PM3B	A09	L-Asparagine	N-Source, amino acid	5.3200	0.0000	10.9250	0.6435	12.0250	0.2475
PM3B	A10	L-Aspartic acid	N-Source, amino acid	6.1350	1.3364	7.1200	0.4667	7.0700	0.4950
PM3B	A11	L-Cysteine	N-Source, amino acid	0.7050	0.0778	4.6500	0.3536	5.6750	0.1626
PM3B	A12	L-Glutamic acid	N-Source, amino acid	5.2800	0.6223	9.9650	0.2333	7.3800	0.7495
PM3B	B01	L-Glutamine	N-Source, amino acid	9.5900	0.3111	14.7000	0.6081	21.5650	0.3748
PM3B	B02	Glycine	N-Source, amino acid	1.7350	0.2192	2.1850	0.0212	3.1250	0.0354
PM3B	B03	L-Histidine	N-Source, amino acid	10.9450	0.3465	13.3550	0.4455	18.6800	1.3435
PM3B	B04	L-Isoleucine	N-Source, amino acid	2.9600	0.3960	1.3850	0.0354	3.6600	0.4808
PM3B	B05	L-Leucine	N-Source, amino acid	4.3900	0.1414	9.6950	0.4172	9.9050	0.1909
PM3B	B06	L-Lysine	N-Source, amino acid	0.9650	0.3041	8.8200	0.7495	10.7550	0.0919
PM3B	B07	L-Methionine	N-Source, amino acid	0.8600	0.2970	0.5750	0.1202	1.2750	0.2192
PM3B	B08	L-Phenylalanine	N-Source, amino acid	4.3300	0.0849	7.6600	1.0465	9.1750	0.2051
PM3B	B09	L-Proline	N-Source, amino acid	1.2050	0.0495	0.8900	0.4950	1.2250	0.0212
PM3B	B10	L-Serine	N-Source, amino acid	0.4350	0.0778	0.5400	0.0707	0.9850	0.0212
PM3B	B11	L-Threonine	N-Source, amino acid	0.9850	0.0636	0.5000	0.1131	1.8400	0.4384
PM3B	B12	L-Tryptophan	N-Source, amino acid	3.9550	0.9546	9.6800	0.2263	9.7400	0.3111
PM3B	C01	L-Tyrosine	N-Source, amino acid	2.0050	0.1485	6.7450	1.1809	8.1800	0.1556
PM3B	C02	L-Valine	N-Source, amino acid	2.6800	0.1273	1.6400	0.3818	3.2050	0.0354
PM3B	C03	D-Alanine	N-Source, amino acid	2.3150	0.1202	2.5050	0.0071	3.0550	0.1485
PM3B	C04	D-Asparagine	N-Source, amino acid	2.3050	0.2051	1.1150	0.2475	2.6500	0.0283
PM3B	C05	D-Aspartic acid	N-Source, amino acid	2.5650	0.3606	1.0350	0.3041	2.6050	0.1626
PM3B	C06	D-Glutamic acid	N-Source, amino acid	1.4700	0.0283	0.7500	0.0283	1.7850	0.3889
PM3B	C07	D-Lysine	N-Source, amino acid	4.1600	0.0990	7.9000	0.5798	13.2550	0.3889
PM3B	C08	D-Serine	N-Source, amino acid	1.5300	0.0849	1.0200	0.3253	1.5950	0.0495
PM3B	C09	D-Valine	N-Source, amino acid	1.4350	0.2758	0.6200	0.0283	1.5050	0.0636
PM3B	C10	L-Citrulline	N-Source, amino acid	1.9800	0.4950	8.8800	0.1980	6.6850	0.1626
PM3B	C11	L-Homoserine	N-Source, amino acid	1.7900	0.0990	1.2300	0.2404	2.5250	0.2051
PM3B	C12	L-Ornithine	N-Source, amino acid	0.9500	0.0424	6.2250	0.1202	10.7650	0.0495
PM3B	D01	N-Acetyl-L-Glutamic acid	N-Source, amino acid	1.0500	0.1414	0.7550	0.1626	1.3500	0.1131
PM3B	D02	N-Phthaloyl-L-Glutamic acid	N-Source, amino acid	1.3250	0.0495	0.5600	0.0849	1.5850	0.0636
PM3B	D03	L-Pyroglutamic acid	N-Source, amino acid	1.8600	0.2828	0.8550	0.0212	2.4800	0.0141
PM3B	D04	Hydroxylamine	N-Source, other	0.2100	0.0424	0.1600	0.0000	0.5500	0.5657
PM3B	D05	Methylamine	N-Source, other	2.4250	0.1485	5.5700	0.1838	12.8100	1.1031
PM3B	D06	N-Amylamine	N-Source, other	5.3100	0.3536	3.2650	0.2192	8.5700	0.2263
PM3B	D07	N-Butylamine	N-Source, other	5.3600	0.1697	6.0150	0.5869	9.3600	0.0990
PM3B	D08	Ethylamine	N-Source, other	4.4850	0.4031	5.9900	0.3818	10.4950	0.0212
PM3B	D09	Ethanolamine	N-Source, other	4.5300	0.0990	5.6150	0.2192	9.8300	0.2121
PM3B	D10	Ethylenediamine	N-Source, other	0.3300	0.0566	0.3600	0.0566	0.8700	0.6788
PM3B	D11	Putrescine	N-Source, other	6.7200	0.5940	9.6900	0.1697	11.2250	0.3889
PM3B	D12	Agmatine	N-Source, other	2.1900	0.7637	4.2900	0.0707	2.7500	0.0424
PM3B	E01	Histamine	N-Source, other	4.8000	0.2687	6.3500	0.0424	13.4200	0.1414
PM3B	E02	b-Phenylethylamine	N-Source, other	3.3050	0.9405	0.8400	0.1697	8.1300	0.0424
PM3B	E03	Tyramine	N-Source, other	3.9900	1.0465	4.5700	1.1738	5.5400	0.2970
PM3B	E04	Acetamide	N-Source, other	4.3950	0.0636	9.4250	0.1485	21.0850	1.0677
PM3B	E05	Formamide	N-Source, other	9.4750	0.4313	9.3900	0.0707	17.8400	1.3718
PM3B	E06	Glucuronamide	N-Source, other	8.0950	0.2758	7.1550	0.4313	17.3200	0.0424
PM3B	E07	D,L-Lactamide	N-Source, other	8.1400	1.3718	7.6200	0.3677	8.4600	0.4384
PM3B	E08	D-Glucosamine	N-Source, other	0.3850	0.0495	0.3250	0.0212	2.2050	2.7365
PM3B	E09	D-Galactosamine	N-Source, other	9.1050	0.1061	4.4950	0.0495	12.7250	0.0071
PM3B	E10	D-Mannosamine	N-Source, other	1.6450	0.0919	1.0500	0.0141	13.6450	0.8839
PM3B	E11	N-Acetyl-D-Glucosamine	N-Source, other	1.2050	0.1202	0.5250	0.1626	1.2000	0.1697
PM3B	E12	N-Acetyl-D-Galactosamine	N-Source, other	0.9150	0.1909	0.4200	0.0707	0.8800	0.1131
PM3B	F01	N-Acetyl-D-Mannosamine	N-Source, other	0.9850	0.0071	0.5400	0.0283	1.2250	0.0071
PM3B	F02	Adenine	N-Source, other	7.2600	0.3111	5.7950	0.5869	8.7800	0.8627
PM3B	F03	Adenosine	N-Source, other	4.4650	0.9405	0.1850	0.0212	3.1050	1.2092
PM3B	F04	Cytidine	N-Source, other	2.0350	0.2192	1.1250	0.1909	2.9900	0.4525
PM3B	F05	Cytosine	N-Source, other	7.6550	0.0071	7.9050	0.2475	10.1500	0.8061
PM3B	F06	Guanine	N-Source, other	4.0500	4.3841	0.9450	0.0495	12.0400	14.7502
PM3B	F07	Guanosine	N-Source, other	0.5950	0.0071	0.5700	0.0141	1.0900	0.0849
PM3B	F08	Thymine	N-Source, other	2.1250	0.1909	8.6300	0.8344	14.3700	0.2687
PM3B	F09	Thymidine	N-Source, other	1.3400	0.0283	0.6600	0.1556	1.5200	0.0283
PM3B	F10	Uracil	N-Source, other	1.3700	0.0424	0.7050	0.0495	1.6450	0.4596
PM3B	F11	Uridine	N-Source, other	0.8950	0.0071	0.3950	0.0778	1.1150	0.0919
PM3B	F12	Inosine	N-Source, other	1.0400	0.1131	0.3800	0.0283	0.9550	0.0071
PM3B	G01	Xanthine	N-Source, other	0.0550	0.0354	0.2900	0.2687	1.2800	0.4525
PM3B	G02	Xanthosine	N-Source, other	1.2350	0.0354	0.6550	0.0636	1.4050	0.0354
PM3B	G03	Uric acid	N-Source, other	7.1300	0.5657	6.7900	0.4101	7.0650	0.3606
PM3B	G04	Alloxan	N-Source, other	3.9900	0.0849	3.5300	0.1980	3.7700	0.1273
PM3B	G05	Allantoin	N-Source, other	5.3550	0.2475	3.5600	0.6647	5.2950	0.1909
PM3B	G06	Parabanic acid	N-Source, other	6.5200	0.0707	7.9300	0.2121	12.3300	0.0283
PM3B	G07	D,L-a-Amino-N-Butyric acid	N-Source, other	4.5600	0.8910	1.3400	0.7495	5.2150	0.1485
PM3B	G08	g-Amino-N-Butyric acid	N-Source, other	1.1550	0.1061	1.2450	0.0071	1.7500	0.3818
PM3B	G09	e-Amino-N-Caproic acid	N-Source, other	1.2150	0.0212	6.0100	0.4525	11.7900	0.2546
PM3B	G10	D,L-a-Amino-Caprylic acid	N-Source, other	0.5150	0.0636	0.2700	0.0990	0.5450	0.5445
PM3B	G11	d-Amino-N-Valeric acid	N-Source, other	0.8500	0.0566	6.0950	0.6435	9.7800	0.8627
PM3B	G12	a-Amino-N-Valeric acid	N-Source, other	3.1050	1.0112	6.7900	0.4384	6.3300	0.6930
PM3B	H01	Ala-Asp	N-Source, peptide	1.6900	0.1414	0.3950	0.0212	0.8300	0.0141
PM3B	H02	Ala-Gln	N-Source, peptide	8.0350	0.1061	10.0550	0.9970	6.1300	0.0990
PM3B	H03	Ala-Glu	N-Source, peptide	3.7000	0.0000	0.8500	0.0566	1.2850	0.1909
PM3B	H04	Ala-Gly	N-Source, peptide	2.4300	0.0707	3.5850	0.3465	2.4900	0.1980
PM3B	H05	Ala-His	N-Source, peptide	6.5650	0.6435	6.8850	0.7990	4.2550	0.5586
PM3B	H06	Ala-Leu	N-Source, peptide	4.1450	0.1202	4.2900	0.3536	3.9400	0.4525
PM3B	H07	Ala-Thr	N-Source, peptide	1.4750	0.1202	0.3850	0.0495	3.2350	0.1626
PM3B	H08	Gly-Asn	N-Source, peptide	2.4750	0.0071	0.9700	0.0990	1.7600	0.2404
PM3B	H09	Gly-Gln	N-Source, peptide	4.2250	0.0495	5.5100	0.4101	2.7350	0.1768
PM3B	H10	Gly-Glu	N-Source, peptide	2.0700	0.0566	0.4400	0.0000	0.7150	0.0495
PM3B	H11	Gly-Met	N-Source, peptide	2.4350	0.3323	3.6400	0.1838	3.9150	0.0212
PM3B	H12	Met-Ala	N-Source, peptide	1.9150	0.0354	3.0350	0.1626	3.6700	0.0707
